# A Gait Analysis in Professional Dancers: A Cross-Sectional Study

**DOI:** 10.3390/bioengineering11111102

**Published:** 2024-10-31

**Authors:** Manghe Fidelis Obi, Walther Gina, Tarun Goswami

**Affiliations:** 1 Department of Biomedical, Industrial and Human Factors Engineering, Wright State University, Dayton, OH 45435, USA; manghe.2@wright.edu; 2 School of Fine and Performing Arts, Wright State University, Dayton, OH 45435, USA; gina.walther@wright.edu

**Keywords:** gait, professional dancers, non-dancers, angular velocity, injury prevention, velocity, acceleration

## Abstract

A gait analysis serves as a critical tool for examining the biomechanical differences in movement patterns between trained and untrained individuals. This study investigates the nuanced differences in gait patterns between professional ballet dancers and non-dancers, with a focus on angular velocities and accelerations across key body joints. By analyzing the positions and movements of the head, neck, shoulder, spine, hip, knee, and ankle in both the sagittal (SP) and frontal (FP) planes, the study aims to identify key distinctions in joint dynamics that arise from differing levels of physical training. The study involved ten participants in total, comprising four professional female ballet dancers and six non-dancer students (three males and three females). In the first experiment, participants performed walking trials at five different speeds, while in the second experiment, the ballet dancers performed six distinct dance routines. Data were captured using a Kinetics motion capture camera system, which recorded the maximum and minimum angular velocities and accelerations during both walking and dancing. Key findings reveal significant differences in joint dynamics. For example, non-dancers’ right shoulder exhibited a maximum angular velocity of −0.47 rad/s and a minimum of 0.71 rad/s, while dancers showed a maximum of −0.09 rad/s and a minimum of 0.07 rad/s. The right knee also displayed notable differences, with angular velocities ranging from −3.88 rad/s to 2.61 rad/s for non-dancers, compared to −0.35 rad/s to 0.54 rad/s for dancers. In terms of acceleration, dancers’ left shoulder reached a maximum of 3.952 mm/s^2^, while their right shoulder had a minimum of −0.1 mm/s^2^. For non-dancers, the left elbow showed a maximum acceleration of 2.997 mm/s^2^, while the right elbow had a minimum of 0.05 mm/s^2^. These variations in angular velocity and acceleration underscore the distinct roles and movements of various joints, highlighting differences in muscle coordination and joint control. Understanding these patterns is crucial for assessing joint function, optimizing training, and developing intervention strategies for injury prevention and rehabilitation. The findings hold significant implications for the dance community and other physically active populations, offering valuable insights into performance enhancement and movement health.

## 1. Introduction

In biomechanics, a gait analysis is a fundamental method that measures stride length, joint angles, ground response forces, and temporal spatial properties to evaluate human locomotion patterns [1]. Because of this, researchers may assess therapies, comprehend variations from normal gait, and investigate the mechanics underlying different musculoskeletal disorders. Renowned for their exceptional precision, control, and coordination, professional dancers go through rigorous physical training that leads to special gait patterns [2]. The biomechanics of dance movements have been the subject of several studies, but little of that research has been exclusively devoted to analyzing the gait of professional dancers [3,4]. A gait analysis in dancers offers a holistic view of movement beyond choreographed routines, providing insights into how dance training affects overall gait mechanics. By studying parameters like stride length, joint angles, and ground reaction forces, researchers can investigate the impact of years of dance training on movement efficiency and injury resilience [5,6]. However, there remains a gap in understanding the full biomechanical adaptations in gait among professional dancers compared to non-dancers.

This study compares the gait patterns of professional dancers versus non-dancers, with an emphasis on ground response forces, joint kinematics, and temporal spatial characteristics [7,8]. Professional dancers are thought to have more controlled and efficient gait mechanics than non-dancers because of their considerable training; these mechanics include longer strides, more stable joint angles, and optimal force distribution [9]. To further promote multidisciplinary cooperation, this study was carried out as a student-driven partnership between the dance and engineering departments. Students were able to gather insights and experiment with novel approaches due to the project’s pilot characteristics, which established a solid basis for future, more extensive research. We are confident that this collaboration has yielded meaningful preliminary results that will inform larger-scale research with more rigorous statistical analyses in the future.

### 1.1. Related Work

Prior studies have mostly examined gait analyses in clinical settings, focusing on patients with neurological problems, musculoskeletal disorders [10,11], or anomalies in gait [12,13]. In contrast, little research has been performed on the gait patterns of dancers, especially those who perform professionally. Nonetheless, a few studies have investigated the biomechanical elements of dance moves and methods. In their analysis of the kinematic and kinetic properties of ballet jumps, for example, Smith et al. [14,15] emphasized the significance of ankle and knee joint movements for peak performance. The impact of various ballet styles on lower extremity muscle activation patterns during jumps was also examined by Davis et al. [13,16]. In addition to these dance-related studies, there has been notable progress in gait analyses in other areas. An algorithm was developed to correct knee flexion axis errors in gait data, significantly reducing errors and enhancing the certainty and precision of knee kinematics [13]. This PCA-based algorithm surpassed other established algorithms in a healthy subject population. Another study investigated the impact of different anatomical frames on net knee joint moments, revealing significant differences in peak knee moments in the sagittal plane. This underscores the importance of considering anatomical frames in knee joint moment calculations during a gait analysis [17]. Furthermore, a biomechanical study explored the mechanics of running in 13 individuals, observing changes in hip, knee, and ankle joint motions, as well as electromyographic data, with increasing gait speed. The study noted alterations in body mechanics, muscle activity, and joint motions as the speed of gait increased [18]. In the context of fall prevention among the elderly, the research investigated factors influencing Minimum Foot Clearance (MFC), a potential cause of tripping falls. The study found that MFC is highly influenced by the strength of hip flexors and the angle of ankle dorsiflexion, suggesting the importance of considering these factors in fall prevention training for the elderly [15]. While these studies have contributed valuable insights into the mechanics of dance movements and various aspects of a gait analysis, there remains a gap in understanding the comprehensive gait mechanics specific to professional dancers [19,20].

#### Study Design and Objectives

The principal aim of this study is to perform a quantitative gait analysis comparing the gait parameters of professional dancers with those of non-dancers. The specific research objectives are as follows:

1. To determine discrepancies in temporal–spatial parameters, such as stride length, step width, and gait velocity, between professional dancers and non-dancers.
2. To analyze joint kinematics, including joint angles and range of motion across the gait cycle, to identify precise distinctions in joint motion between the two groups.
3. To evaluate ground reaction forces and their components, which will provide insight into the distribution of forces and load patterns experienced by professional dancers in contrast to non-dancers.

By achieving these objectives, the study aims to offer a comprehensive understanding of the intricate gait mechanics that characterize professional dancers, highlighting key distinctions that may underpin their superior performance and injury resilience.

In addition to these biomechanical objectives, the research had several collaborative goals. One of the primary aims was to foster collaboration between students from the biomedical engineering and dance departments, focusing on discretizing joint positions in relation to body movements. Another objective was to assess the applicability of the sensors in capturing different motions, rather than relying solely on an established experimental setup for routine testing.

No demographic data were collected from the participants, and no formal consent was required, as this study was a collaborative educational project between the two departments. The study design had to be completed within a 10-week period, which was insufficient time for Institutional Review Board (IRB) application and approval and the execution of the experiments. Given that the study focused on sensor applicability and not medical data collection, the IRB application was deemed unnecessary.

The results presented here are based on the initial data collection. Publishing these findings will lend credibility to future proposals and help secure funding for a statistically designed project that can further explore the capabilities of the equipment used. This initial collaboration and the gathered data will serve as a foundation for future research and more comprehensive testing, while showcasing the potential of interdisciplinary cooperation between the fields of engineering and dance.

## 2. Methodology

This section presents the detailed methodology used in our study, which focuses on a detailed examination of gait dynamics in two different groups: non-dancers as a control group and professional dancers. Participants were carefully matched according to age, gender, and body mass index (BMI) to guarantee the validity of our comparative analyses. To isolate the biomechanical differences between the groups and reduce confounding variables, this matching procedure was essential [21].

Using precision-enhancing force plates and cutting-edge motion capture equipment, we were able to carefully record kinematic and kinetic data while walking. Both the temporal and spatial components of gait were thoroughly understood thanks to the motion capture system’s real-time joint movement recording and the force plates’ measurement of ground reaction forces [22]. The combination of these tools facilitated an accurate and robust data collection process.

Following ethical research guidelines, each subject gave their express and informed consent before the start of our tests [4]. After gathering data, we compared gait metrics between the two groups using a strong statistical analysis to find statistically significant differences [17]. Given the goals of the study, a polynomial regression model was chosen for the analysis since it fit our data the best. With this method, we were able to better capture the correlations between variables than we could have with a mere comparison of means, giving us a more thorough understanding of the participants’ movement patterns [23]. Although we did not initially report variables such as anthropometric data, we recognize the importance of including metrics like body mass index (BMI) to offer a more holistic view of the participants’ characteristics. Future revisions will incorporate these variables to enhance the depth of comparative and regression analyses, enabling more comprehensive interpretations of the data.

### Data Analysis

**Figure 1:**
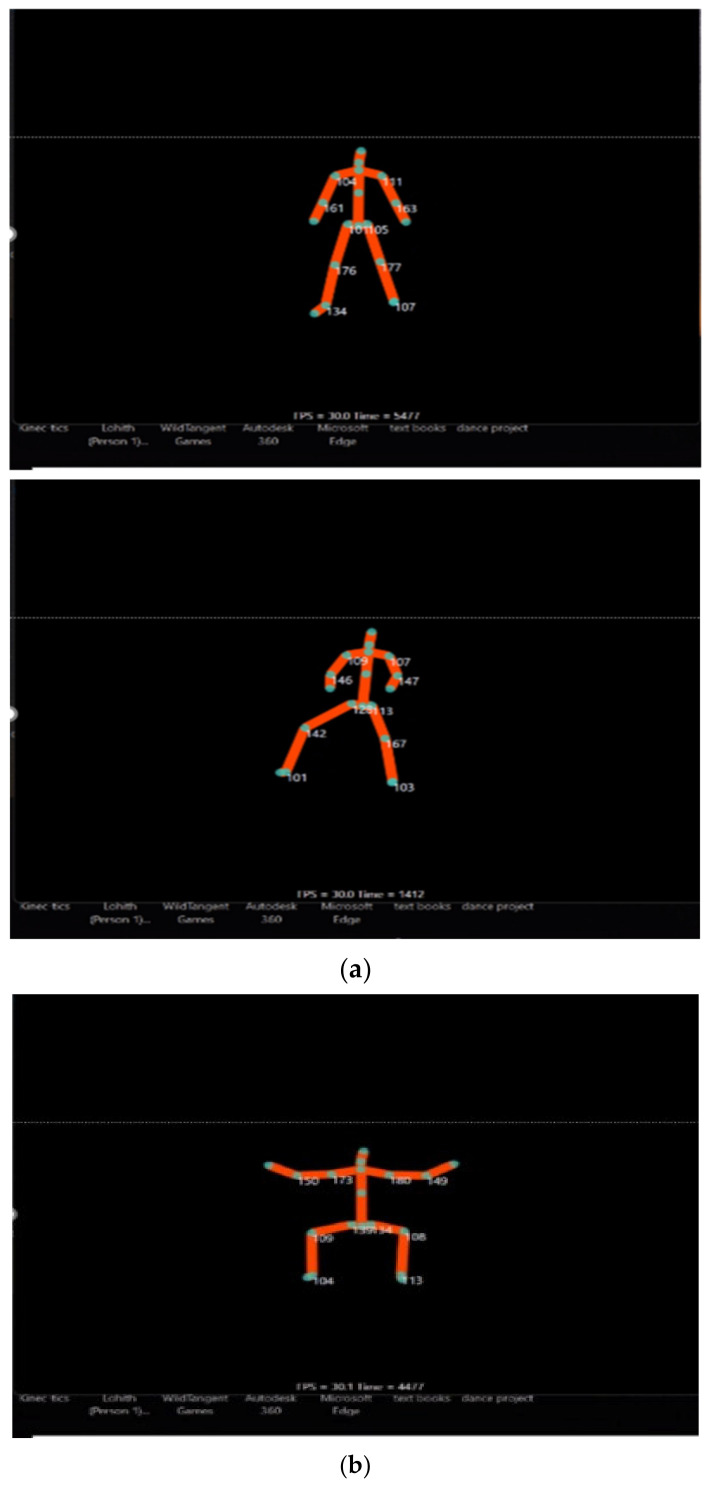
(**a**): Markers detected by the camera during standing. (**b**): Markers detected by the camera during squatting.

The data analysis process in this study began with the identification of key body segments recorded by the Kinetics motion capture system. This system generated two distinct Excel files containing data on both joint angles and positions across three planes: front, sagittal (SP), and transverse. The position file documented upper body joints, including the head, neck, spine, shoulder, elbow, wrist, and hand [13,21], as well as lower body joints such as the hip, knee, ankle, and foot, all recorded in three dimensions (*x*, *y*, *z*). The angle file provided insights into movements of the shoulder and elbow for the upper body [3,16] and the hip, knee, and ankle for the lower body. Figure 1 illustrates the schematic representation of the points detected by the motion capture device [24].

The dependability of the research findings was dependent on two major factors: the camera sensor’s temporal resolution (exposure frequency) and ability to record depth information. The Kinetics motion capture system was utilized to track joint movements in the sagittal (SP) and frontal (FP) planes, with high-speed motion capture being critical for precise data collection, particularly when measuring angular velocities and accelerations.

Unlike previous studies that used dynamic cameras for multi-angle 3D reconstruction, this study concentrated on movement dynamics by evaluating inter-frame variations. This technology allowed for precise tracking of joint motions during walking and dancing routines, providing extensive insights into the differences in gait patterns between professional dancers and non-dancers. The high temporal resolution of the motion capture sensor was especially important for capturing the fast, subtle movements characteristic of dance and walking. This high-frequency capture ensured that even the smallest movements were recorded with precision, facilitating a comprehensive analysis of joint kinematics. Furthermore, the system’s ability to capture depth information enhanced the data’s reliability by allowing for a 3D analysis of joint positions, angles, and velocities.

To better model and interpret the captured data, the study employed polynomial regression equations and R-squared values to describe the relationship between velocity and acceleration across various joints. These statistical models provided an additional layer of reliability by mathematically fitting the motion data into well-defined patterns. The combination of high temporal resolution, depth capture, and precise modeling offered a robust framework for analyzing gait dynamics.

This approach enabled the study to uncover nuanced differences in joint movements, leveraging inter-frame differences to capture the unique motion dynamics of dancers and non-dancers. The integration of these methods ensured the reliability of the findings, allowing for detailed comparisons of joint function, making the results highly relevant for both biomechanical analyses and practical applications such as performance enhancement or rehabilitation.

When angular velocity was calculated, the Kinetics system tracked the positions of body parts such as the shoulder, hip, knee, and ankle in real time, using multiple cameras to capture movement in two or three dimensions, depending on whether depth information was available. Reflective markers placed on the body allowed the system to track the x, y, and z coordinates of joints over time in the sagittal and frontal planes.

The system continuously recorded positional data at a high temporal resolution, capturing the precise position of each joint at every time frame. Angular velocity was calculated from the rate of change of joint angles between successive frames, using the formula

Angular velocity = Δθ/Δt.

In this case, Δt is the time gap between consecutive frames, and Δθ is the change in angle between them.

Integrated algorithms in the system’s software compute important kinematic parameters including acceleration, angular velocity, and joint angles automatically. Instead of being manually entered, these attributes were produced by the system’s integrated computational techniques, which examined inter-frame variations. To enable a more accurate interpretation of the data, a further statistical analysis, such as polynomial regression, modeled the link between acceleration and velocity for each joint.

This research endeavor aspires to shed light on the gait mechanics distinctive to professional dancers and unveil the potential implications of dance training on gait patterns. By comparing the gait parameters of professional dancers with those of non-dancers, the study endeavors to discern pivotal differences that might contribute to enhanced performance and injury resilience among dancers. The findings bear substantial implications for injury prevention, rehabilitation, and performance optimization in both the realm of dance and the broader population.

## 3. Results

The maximum and minimum amounts of angular velocity corresponding to dancer and non-dancer moves are given in Table 1. those of the left and right knee from the dancer and non-dancer moves are displayed in Table 1 and Table 2.

### 3.1. The Result Comparing the Right and Left Angular Velocity of the Dancers and Non-Dancers

The graph of angular velocity illustrates the relationship between the right ankle, right knee, and right hip joints, with each joint represented by a different color. By examining the angular velocity over time, we can observe the maximum and minimum values that correspond to normal walking patterns. The graph reveals significant variations among these joints, particularly in the range of angular velocities each joint experience.

The right ankle displays a range of angular velocities, with a maximum of 1.49 rad/s and a minimum of −1.31 rad/s. This range suggests that the right ankle undergoes both forward and backward angular movements during the activity. These movements indicate the ankle’s involvement in key actions such as stepping or pivoting, which require rapid directional changes.

The right knee demonstrates a much wider range of angular velocities, from a high of 2.61 rad/s to a low of −3.88 rad/s. This substantial variation indicates that the right knee is engaged in significant angular movements, likely involving rapid extensions and flexions. These extreme values suggest that the knee plays a critical role in the analyzed activity, undergoing intense dynamic motions, such as bending and straightening during walking.

The right hip shows a more moderate range of angular velocities, with a maximum of 0.83 rad/s and a minimum of −0.85 rad/s. This indicates that the right hip is involved in angular movements, though to a lesser extent than the knee. The hip’s role during this activity appears to be one of steady support and balance, with less intense rotational movements compared to the more dynamic joints like the knee.

The right shoulder exhibits a maximum angular velocity of 0.71 rad/s and a minimum of −0.47 rad/s, indicating moderate angular motion at the shoulder joint. This range suggests that the shoulder experiences some forward and backward movement, though the intensity of the motion is not as pronounced as in the lower limb joints.

Finally, the right elbow demonstrates angular velocities between 1.00 rad/s (maximum) and −1.26 rad/s (minimum). This range indicates forward and backward angular motion at the elbow joint, suggesting that the elbow is engaged in relatively dynamic movements, likely contributing to arm swings or other similar motions during the analyzed activity.

These observations highlight the different roles and intensities of angular movements across the right-side joints during normal walking. The knee stands out with the most significant angular movement, while the hip and shoulder exhibit more moderate motions. Understanding these variations can provide valuable insights into the biomechanics of walking, the function of each joint, and potential areas for targeted training or rehabilitation.

**Figure 2:**
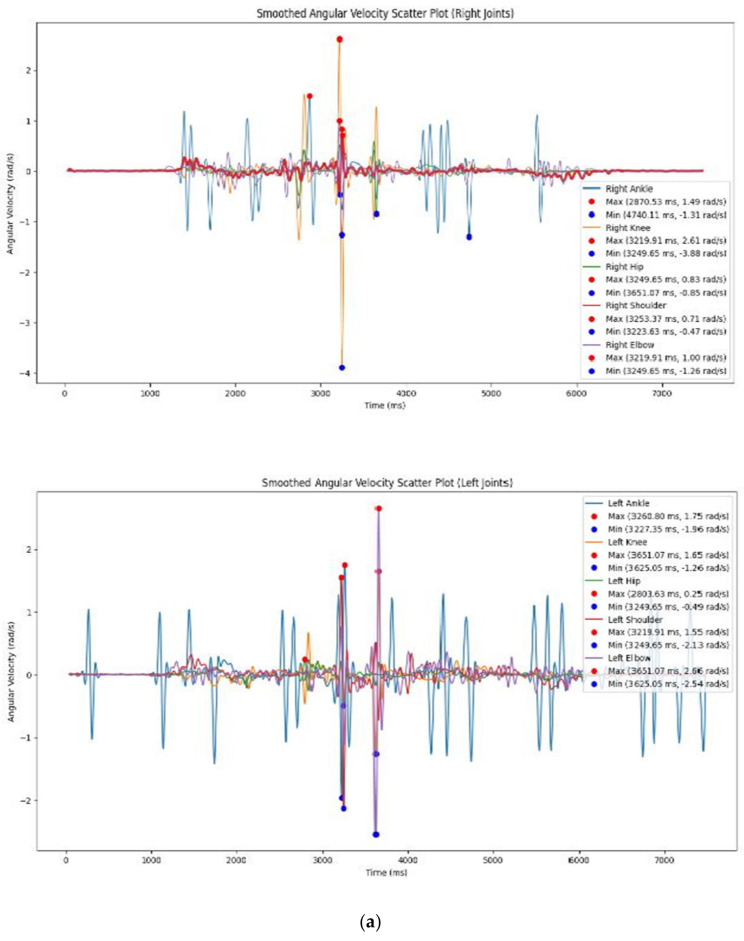

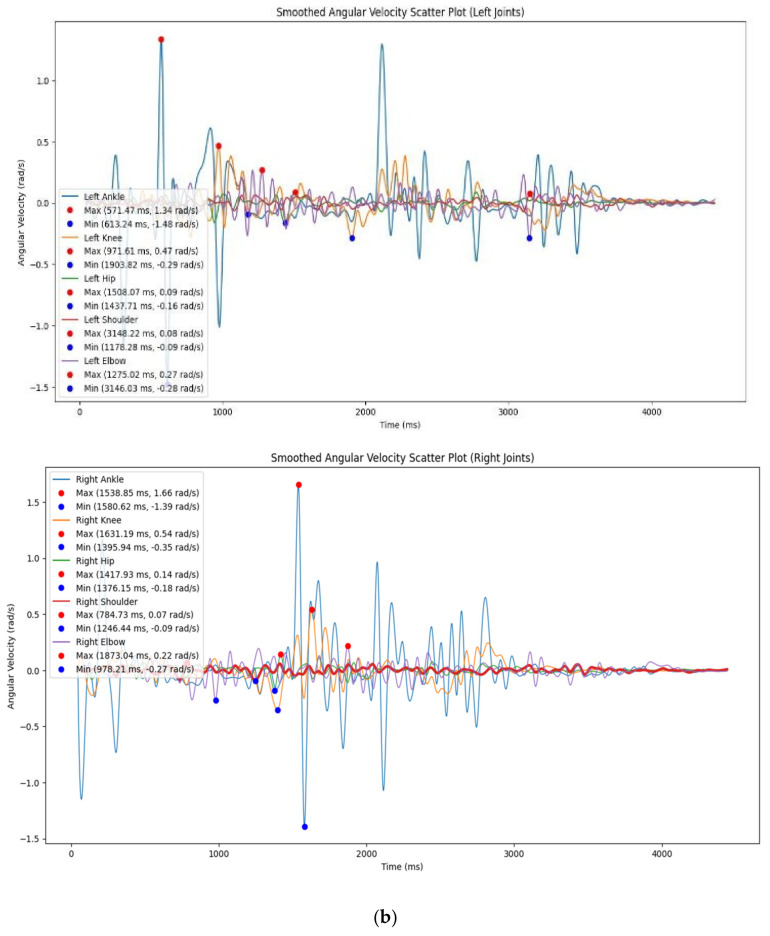
(**a**) Angular velocity of the five joints for the non-dancers’ left and right. (see Figure 2a,b for details). (**b**) Angular velocity of the five joints for the dancers’ left and right.

**Figure 3:**
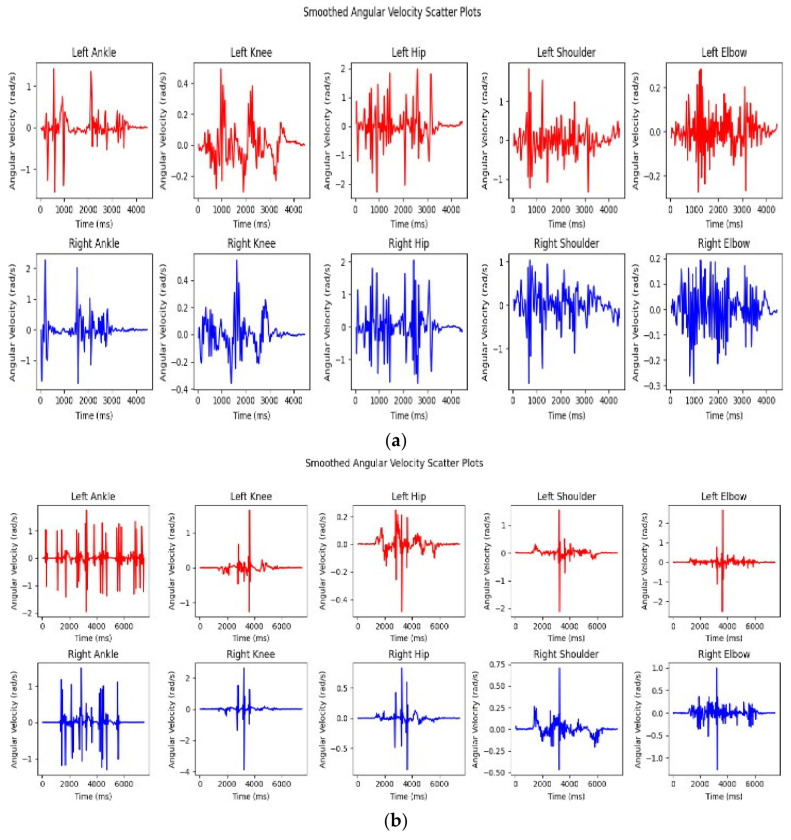
(**a**) Angular velocities of the five joints for the dancers, both left and right. (**b**) Angular velocity of the five joints for the dancers, both left and right.

Key differences in angular velocity are observed, with the right knee showing the most pronounced movements, having the highest maximum angular velocity of 2.61 rad/s and the lowest minimum angular velocity of −3.88 rad/s. This suggests that the right knee undergoes the most significant angular motion during the activity, likely due to its involvement in dynamic actions such as rapid flexion and extension. The right ankle also shows substantial angular movement, though its range is smaller compared to the right knee. The right hip, shoulder, and elbow exhibit comparatively lower angular velocities, indicating less intense movements in these joints during the activity. These variations in angular velocity likely reflect the distinct roles and functions of the joints, highlighting the right knee’s involvement in high-intensity movements, while other joints like the hip or shoulder have more controlled, moderate movements. This understanding of angular velocity patterns is critical for assessing joint function and biomechanics, and it may assist in identifying areas that could benefit from targeted training or rehabilitation interventions. (refer to Figure 2 and Figure 3). 

The scatter plot of angular velocity illustrates the relationship between the left ankle, knee, and hip joints, with each joint represented by a distinct color. By analyzing the graph, which plots angular velocity over time, we can observe the duration of movements during normal walking. The graph reveals significant differences among the joints, such as the left ankle reaching a maximum angular velocity of 1.75 rad/s and a minimum of −1.96 rad/s, indicating a range of forward and backward movements. The left shoulder shows moderate angular motion, with a maximum of 1.55 rad/s and a minimum of −2.13 rad/s, while the left elbow exhibits more substantial angular movement, ranging from 2.66 rad/s to −2.54 rad/s, suggesting significant forward and backward rotation.

When comparing the left and right joints, the ankles on both sides exhibit similar angular velocity patterns, with both demonstrating forward and backward movements. However, the left ankle has a slightly higher maximum angular velocity than the right. The knees show more asymmetry, with the right knee exhibiting higher maximum and more negative minimum angular velocities than the left knee, indicating more pronounced movements during the activity. Similarly, the left elbow demonstrates higher angular velocities than the right elbow, pointing to greater angular motion in the left elbow joint. Conversely, the hips display lower angular velocities, with the left hip showing reduced angular movement compared to the right hip, while the shoulders exhibit similar motion patterns, though the right shoulder has a slightly higher maximum angular velocity than the left.

In particular, the left knee has a maximum angular velocity of 1.65 rad/s and a minimum of −1.26 rad/s, indicating significant flexion and extension during movement. The left hip, however, shows much lower angular velocities, with a maximum of 0.25 rad/s and a minimum of −0.49 rad/s, suggesting relatively minor angular motion compared to other joints.

The differences in angular velocities between the left and right joints likely reflect the variations in their roles during the analyzed activities. Some joints, such as the knees and elbows, exhibit asymmetrical movements, while others, like the ankles and shoulders, display more symmetrical patterns. Understanding these differences is valuable for assessing joint function and biomechanics and may inform training or rehabilitation programs to address any potential imbalances or asymmetries. The Excel table provides a detailed comparison of the angular positions of the left and right ankle, knee, shoulder, hip, and elbow joints, focusing on their maximum and minimum angular velocities, offering key insights into the movement dynamics of non-dancers during these activities.

#### 3.1.1. Regression Equations in the Context of This Study (Velocity)

The regression equations describe the relationship between the movement of specific joints (shoulder, elbow, hip, knee, ankle) in different planes (*x*, *y*, *z*) and the corresponding polynomial regression equations. Each equation is associated with a coefficient (slope) and an R^2^ value, which indicates the goodness of fit for the regression model. Let us analyze the key points from the provided regression equations.

**Figure 4:**
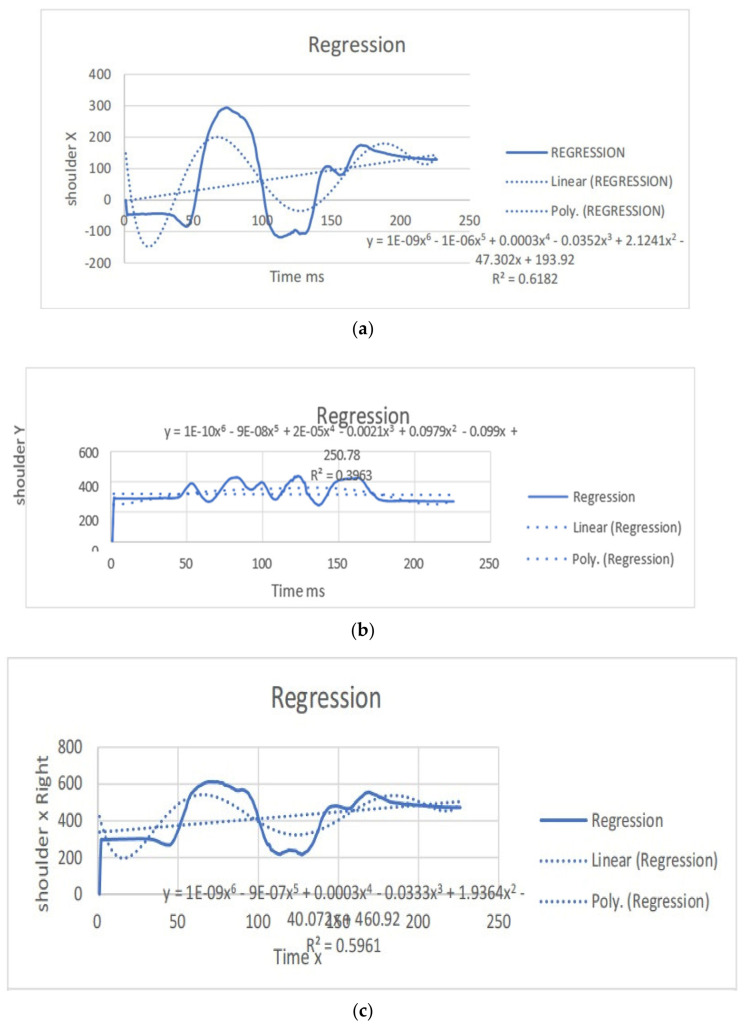

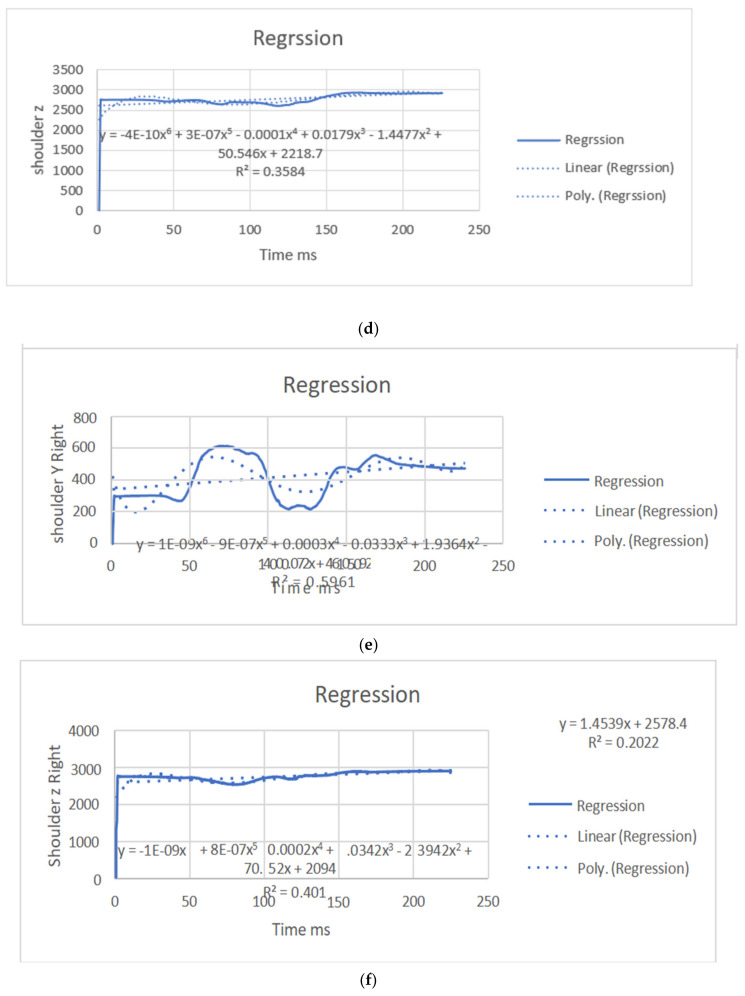
(**a**) Angular positions in the left shoulder x direction with the regression equation line. (**b**) Angular positions in the left shoulder y direction with the regression equation. (**c**) Angular positions in the left shoulder z direction with the regression equation line. (**d**) Angular positions in the right shoulder x direction with the regression equation line. (**e**) Angular positions in the right shoulder y direction with the regression equation line. (**f**) Angular positions in the right shoulder z direction with the regression equation line.

**Figure 5:**
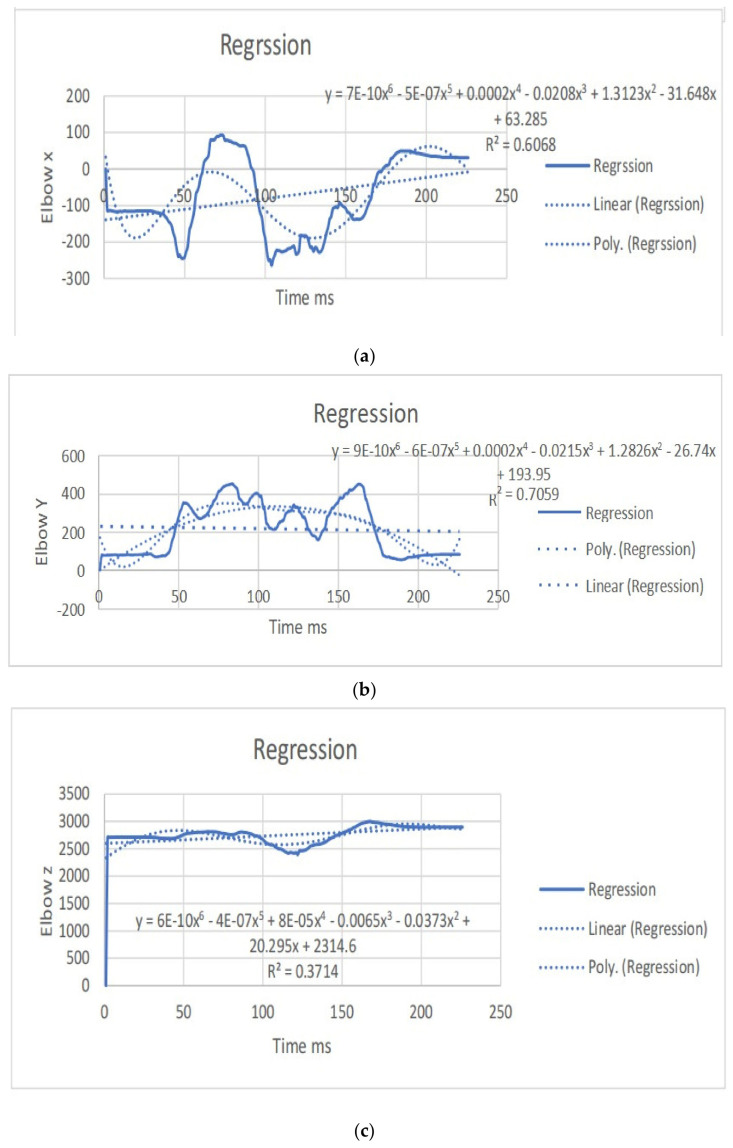

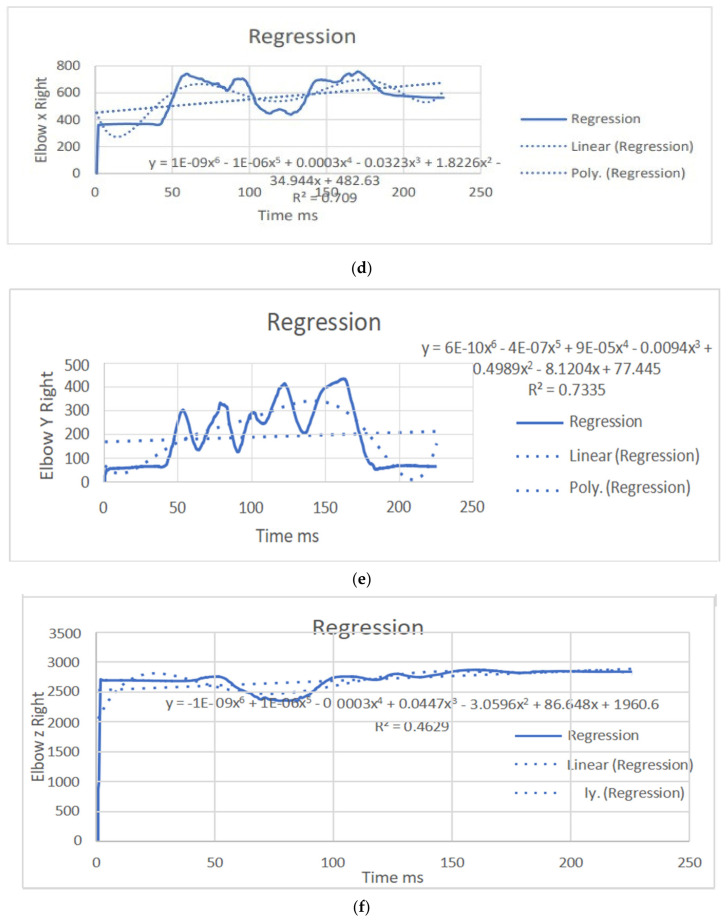
(**a**) Angular positions in the left elbow x direction with the regression equation line. (**b**) Angular positions in the left elbow y direction with the regression equation line. (**c**): Angular positions in the left elbow z direction with the regression equation line. (**d**) Angular positions in the right elbow x direction with the regression equation line. (**e**): Angular positions in the right elbow y direction with the regression equation line. (**f**): Angular positions in the right elbow z direction with the regression equation line.

**Figure 6:**
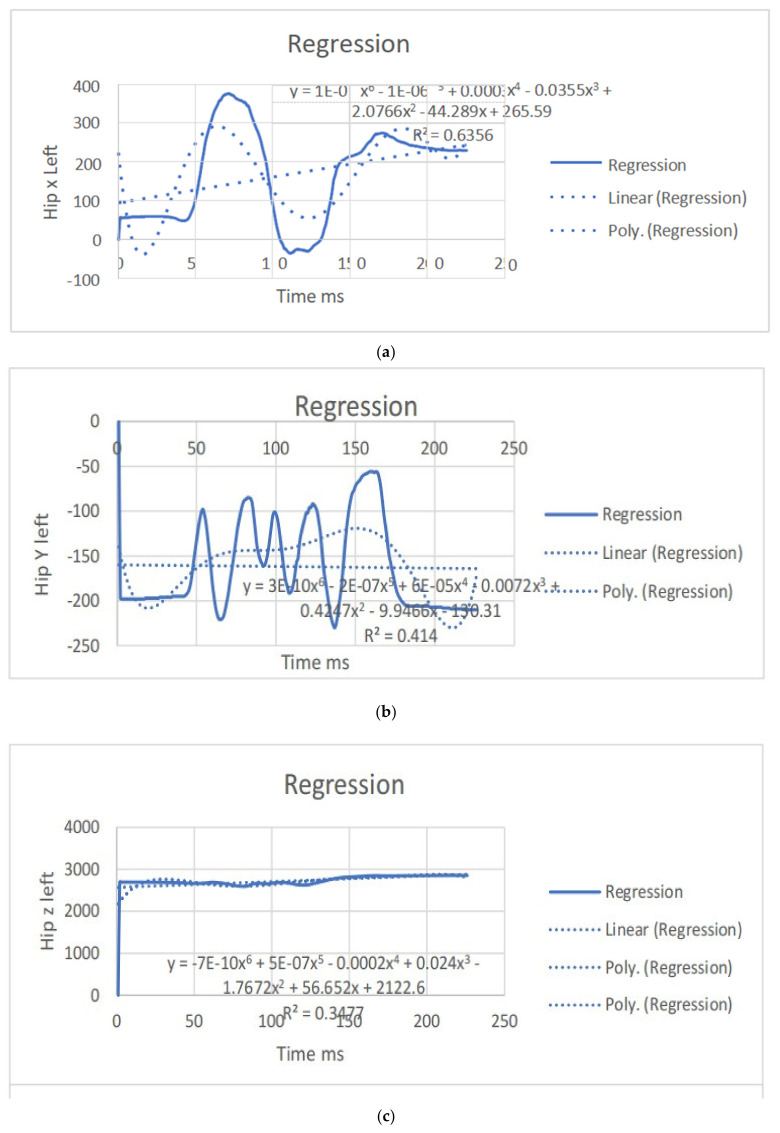

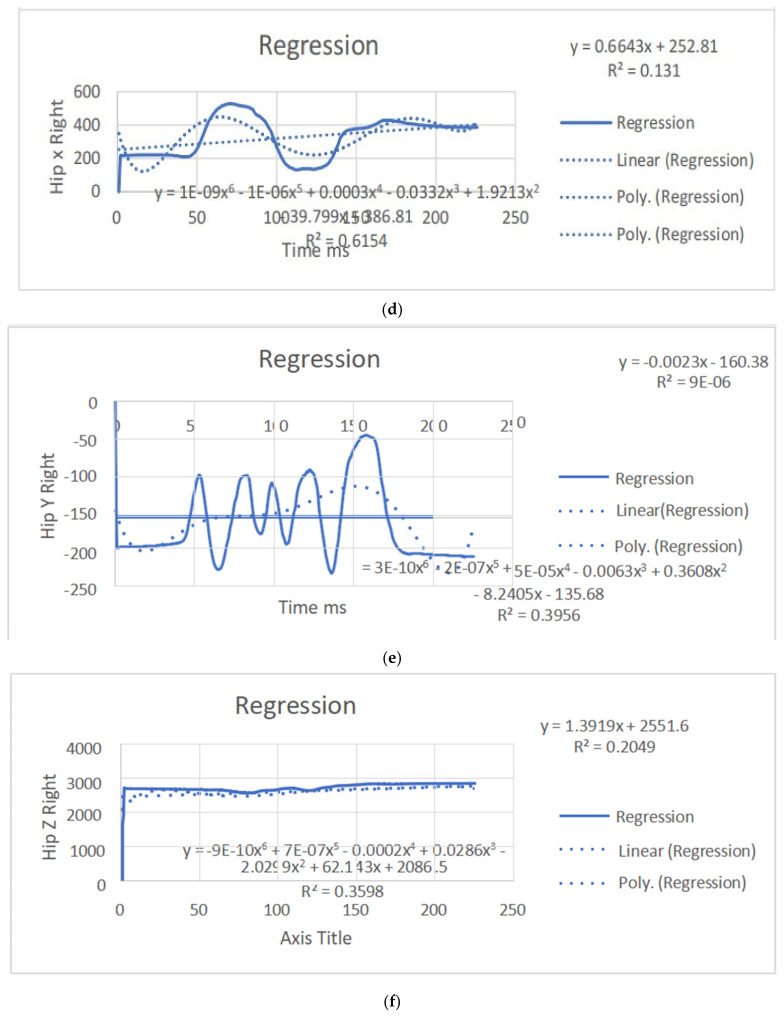
(**a**) Angular positions in the left hip x direction with the regression equation line. (**b**) Angular positions in the left hip y direction with the regression equation line. (**c**) Angular positions in the left hip z direction with the regression equation line. (**d**) Angular positions in the right hip x direction with the regression equation line. (**e**) Angular positions in the right hip y direction with the regression equation line. (**f**): Angular positions in the right hip z direction with the regression equation line.

**Figure 7:**
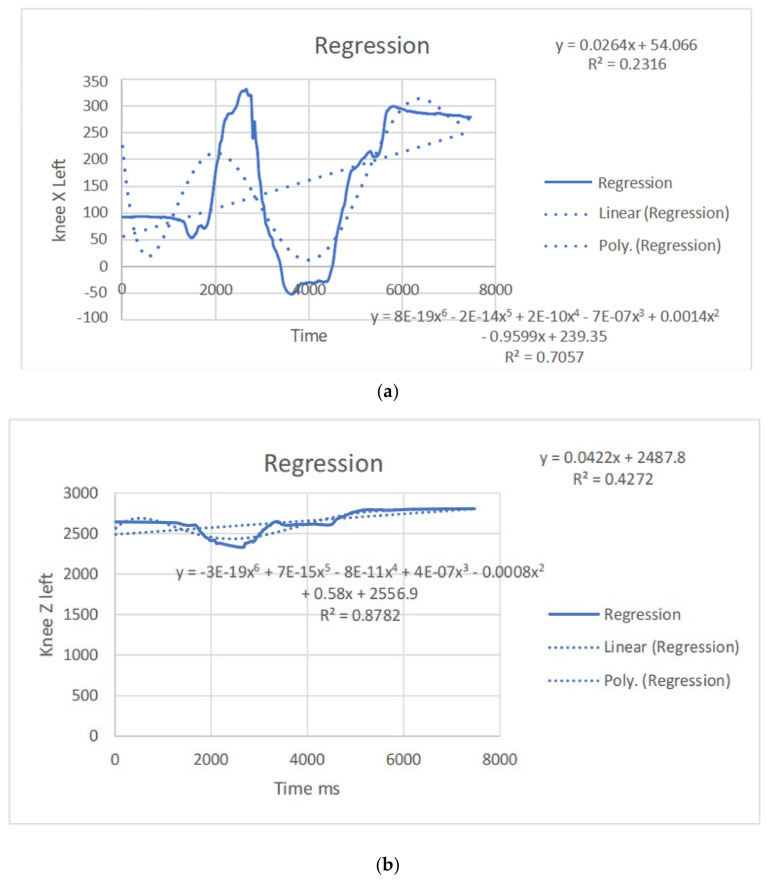

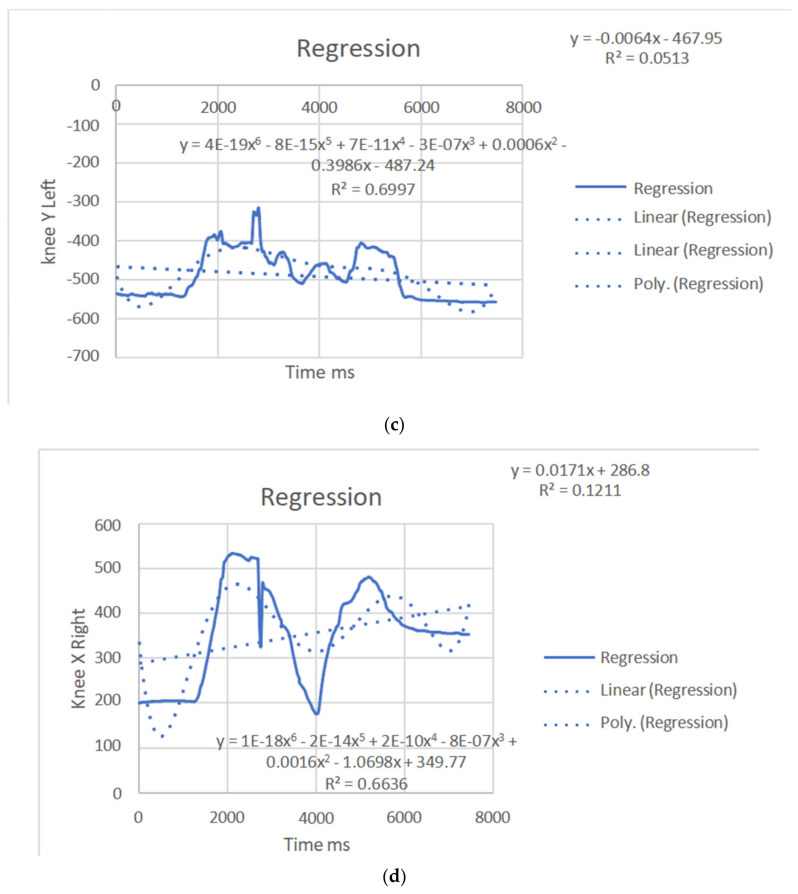
(**a**) Angular positions in the left knee x direction with the regression equation line. (**b**) Angular positions in the left knee y direction with the regression equation line. (**c**) Angular positions in the left knee z direction with the regression equation line. (**d**) Angular positions in the right knee x direction with the regression equation line.

The regression equations provided for different joint movements, such as the shoulder, elbow, hip, knee, and ankle, play a crucial role in understanding the relationship between the independent variable (x, representing time or some other parameter) and the dependent variable (y, representing the movement in a particular direction). Here is a summary of the significance and differences in the regression equations presented: The regression equations for shoulder movements in the x, y, and z directions indicate a complex relationship with time. These equations provide a mathematical model to estimate the position of the shoulder over time during dance moves. Notably, the R^2^ values vary for different directions, suggesting varying degrees of fit. For instance, the R^2^ value for the y direction in the first equation is 0.0012, indicating a weak fit, while the R^2^ value for the x direction in the second equation is 0.6182, indicating a moderate fit. The differences in slopes (coefficients) highlight the unique characteristics of each movement direction, emphasizing the need for separate analyses. The regression equations for elbow movements demonstrate a similar pattern. The equations model the relationship between time and the elbow position in different directions. The R^2^ values vary, indicating the goodness of fit. Notably, the R^2^ value for the y direction in the second equation is 0.0033, suggesting a weak fit, while the R^2^ value for the x direction in the third equation is 0.7335, indicating a strong fit. Differences in slopes reveal distinct patterns of movement in different directions, emphasizing the joint’s multidimensional nature. The regression equations for hip movements provide insights into the relationship between time and hip position in different directions. R^2^ values vary, indicating the variability in the goodness of fit. The differences in slopes highlight the distinct characteristics of each direction. Understanding hip movements is crucial in the context of dance, as the hips play a central role in various dance forms. The regression equations for knee movements model the relationship between time and knee position in different planes. Notably, the R^2^ values vary, with higher values indicating better fits. The significant R^2^ value in the last equation for the z direction (0.8788) suggests a strong relationship. Differences in slopes reflect the multidimensional nature of knee movements during dance. The regression equations for ankle movements highlight the intricate relationship between time and ankle position in different planes. R^2^ values vary, with higher values indicating better fits. The R^2^ value of 0.8088 in the first equation for the z direction suggests a strong fit. Differences in slopes underscore the unique patterns of ankle movement in different directions. The regression equations provide a quantitative approach to understanding the movements of various joints during dance. They offer precise mathematical representations of the relationship between time and joint position. The differences in regression equations for different directions (*x*, *y*, *z*) underscore the multidimensional nature of joint movements. This is crucial for understanding the complexity and nuances of dance motions. The R^2^ values indicate how well the regression equations fit the data. Higher R^2^ values suggest better-fitting models, providing confidence in the accuracy of the equations. Understanding the quantitative aspects of joint movements, as captured by the regression equations, has clinical implications for injury prevention and rehabilitation. Moreover, for dancers and instructors, this information can inform training programs and improve stage performance. The regression equations contribute to the scientific understanding of the biomechanics of dance. They add a quantitative layer to the qualitative observations made in the study, enhancing the overall depth of the analysis. In conclusion, the regression equations presented in the paper serve as a valuable tool for quantifying and understanding the intricate joint movements observed from professional dancers. They contribute significantly to the scientific rigor of the study, offering a mathematical basis for the nuanced analysis of dance motions (Table 3). (refer to Figure 4, Figure 5, Figure 6 and Figure 7). 

#### 3.1.2. Acceleration Showing Maximum and Minimum Points to Various Angle Positions

**Figure 8:**
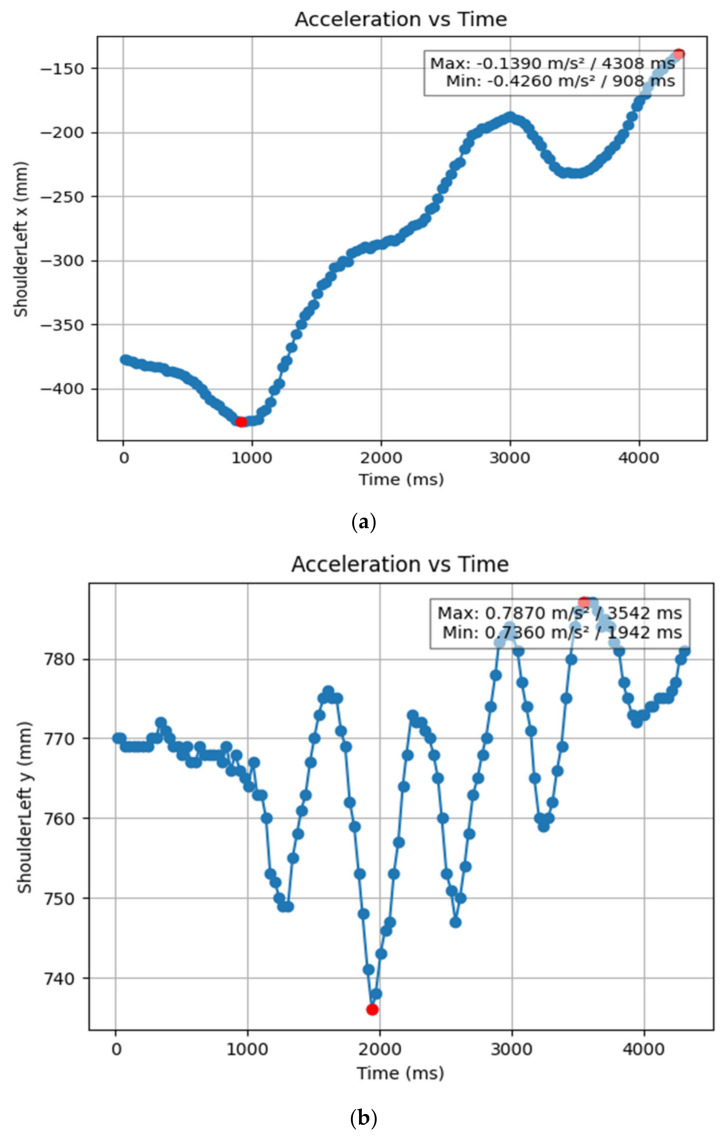

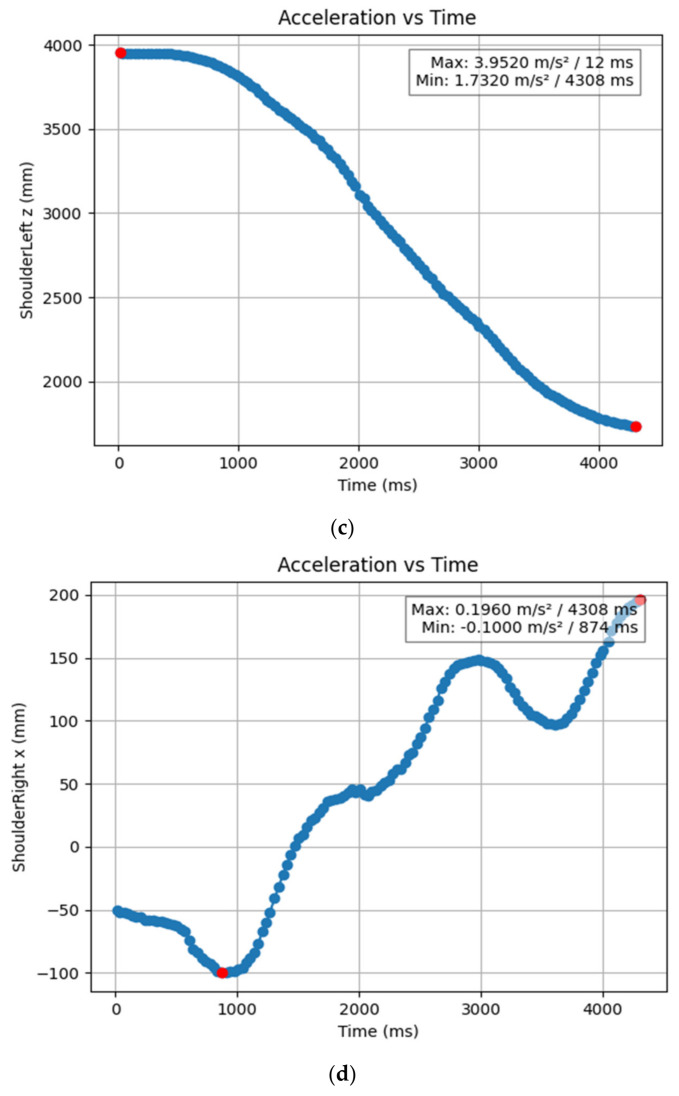

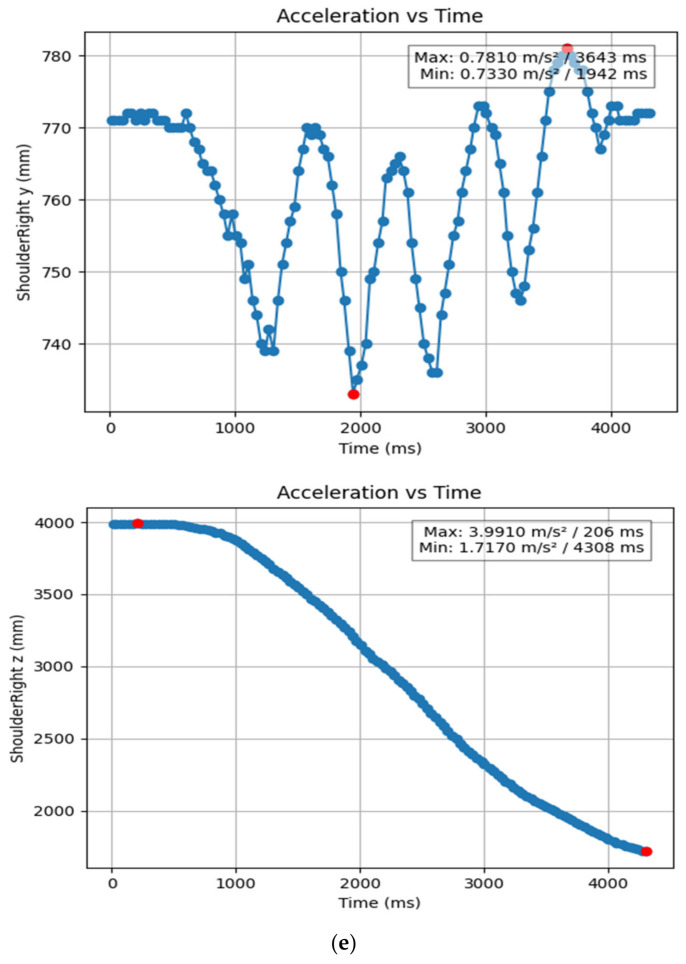
(**a**) Graph acceleration showing maximum and minimum points in the left shoulder x direction angle position. (**b**) Graph acceleration showing maximum and minimum points in the left shoulder y direction angle position. (**c**) Graph acceleration showing maximum and minimum points in the left shoulder z direction angle position. (**d**) Graph acceleration showing maximum and minimum points in the right knee y direction angle position. (**e**) Graph acceleration showing maximum and minimum points in the right shoulder y direction angle position. (**e**) Graph acceleration showing maximum and minimum points in the right shoulder z direction angle position.

**Figure 9:**
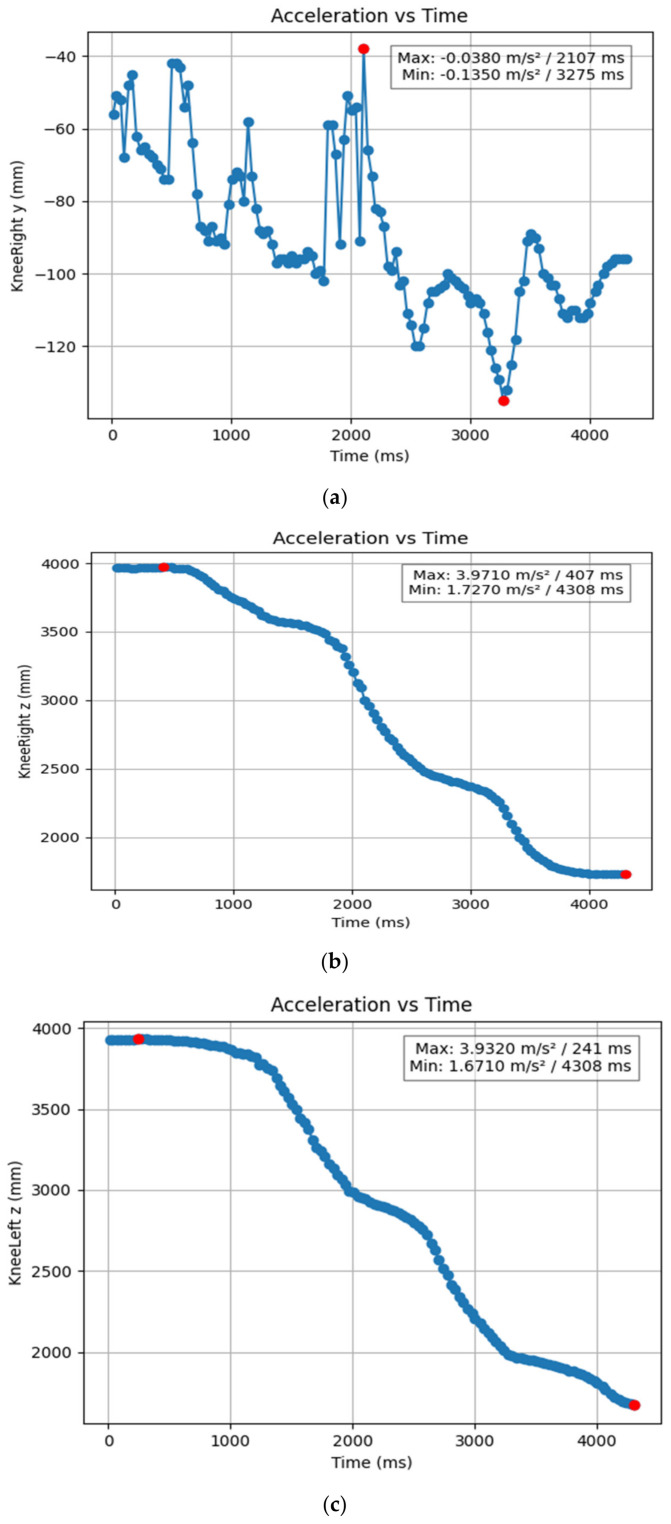

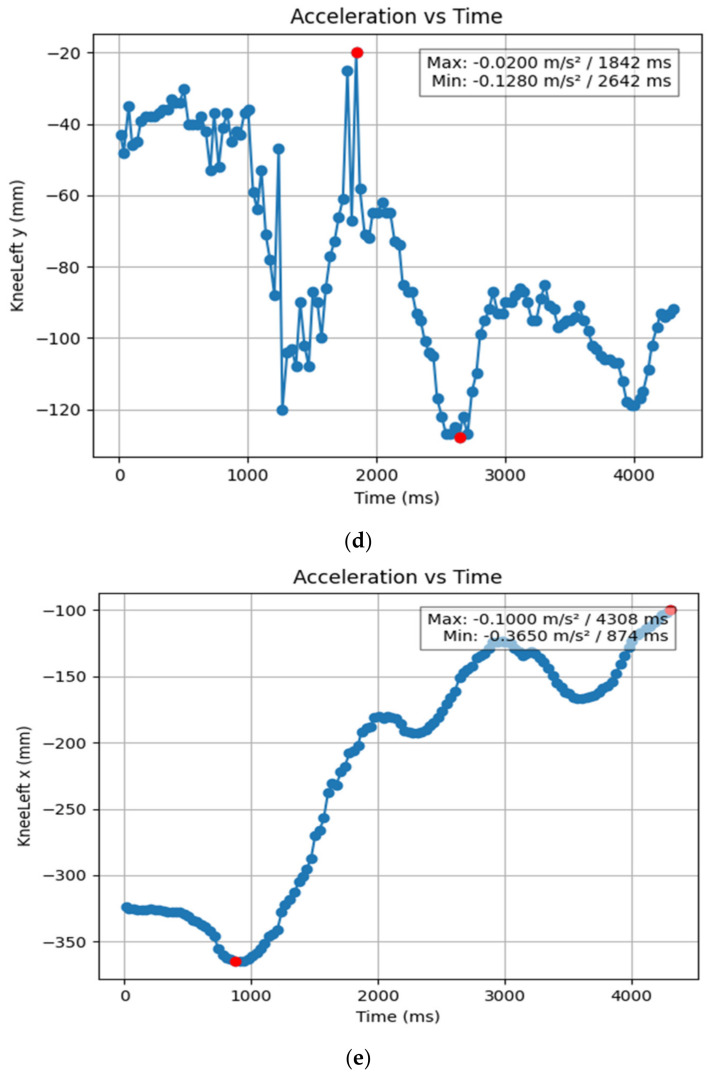
(**a**) Graph acceleration showing maximum and minimum points in the right knee y direction angle position. (**b**) Graph acceleration showing maximum and minimum points in the right knee y direction angle position. (**c**) Graph acceleration showing maximum and minimum points in the knee z direction angle position. (**d**) Graph acceleration showing maximum and minimum points in the left knee y direction angle position. (**e**) Graph acceleration showing maximum and minimum points in the left knee x direction angle position.

**Figure 10:**
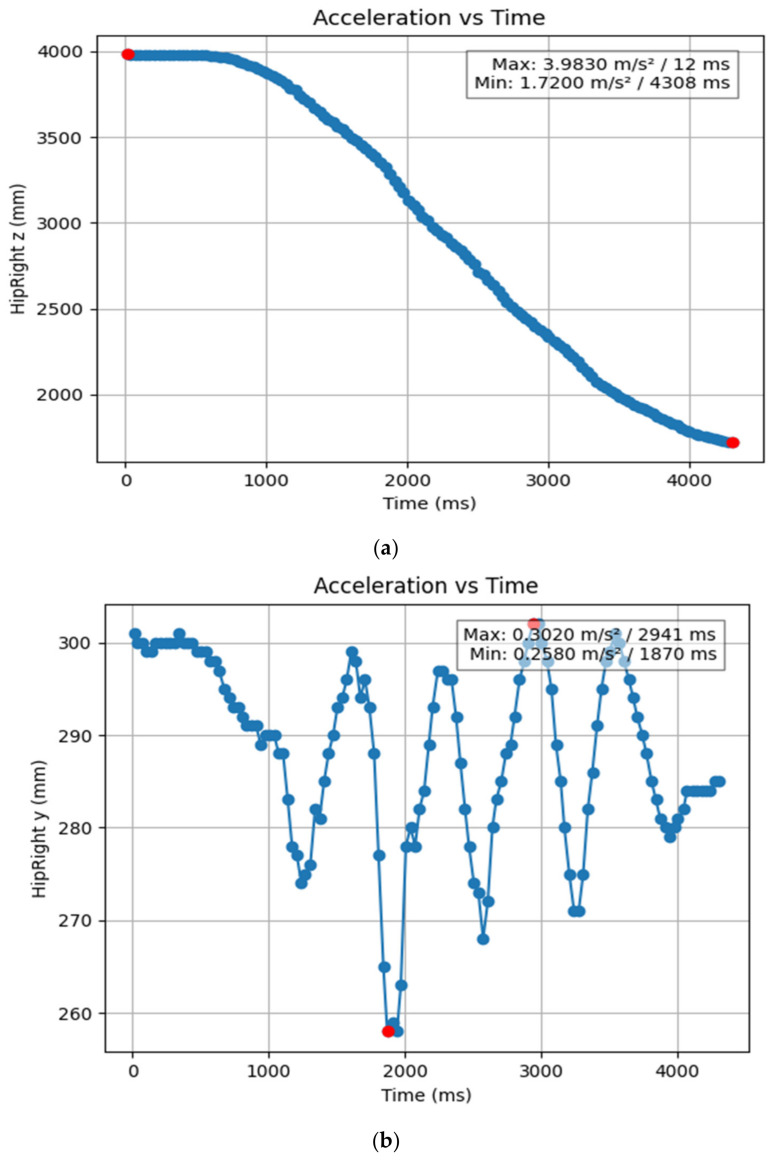

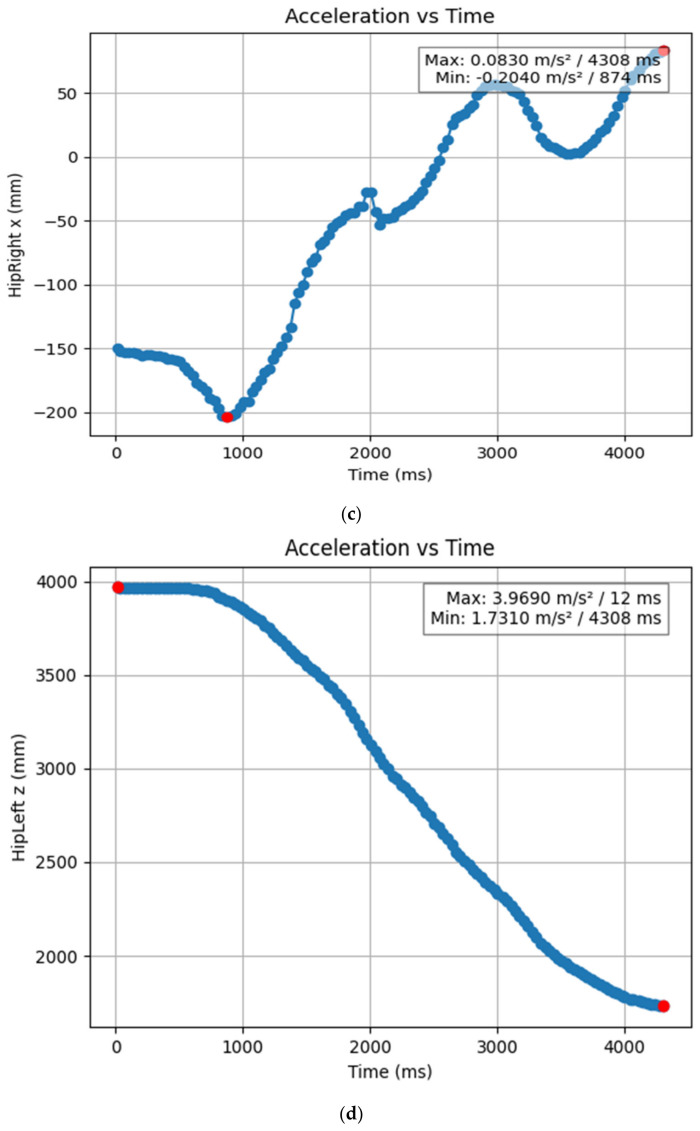

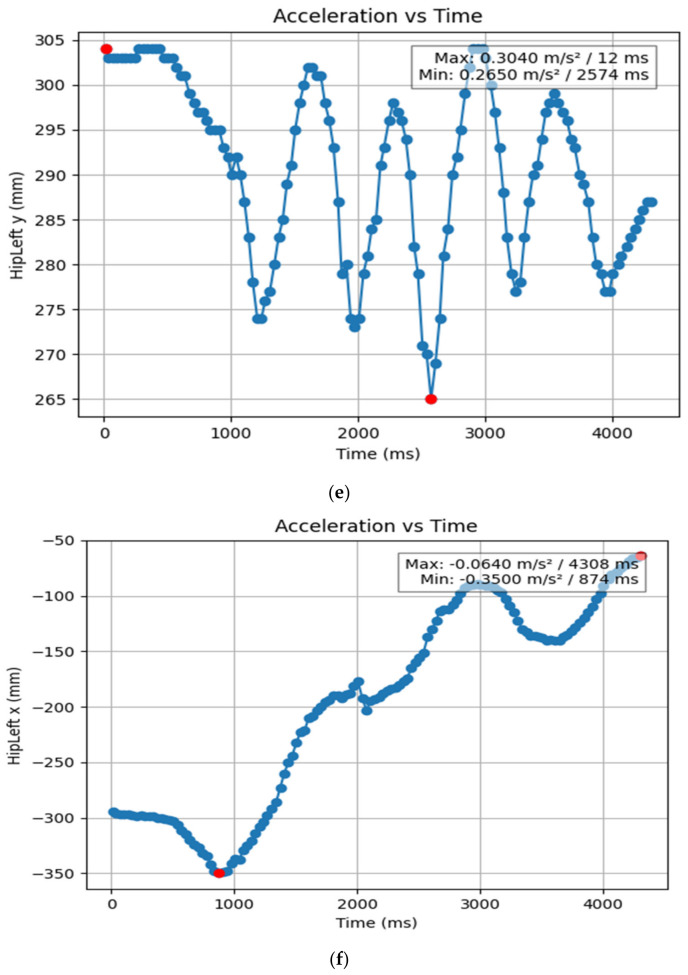
(**a**): Graph acceleration showing maximum and minimum points in the right hip z direction angle position. (**b**): Graph acceleration showing maximum and minimum points in the right hip y direction angle position. (**c**): Graph acceleration showing maximum and minimum points in the right hip x direction angle position. (**d**): Graph acceleration showing maximum and minimum points in the left hip z direction angle position. (**e**): Graph acceleration showing maximum and minimum points in the left hip y direction angle position. (**f**): Graph acceleration showing maximum and minimum points in the left hip x direction angle position.

**Figure 11:**
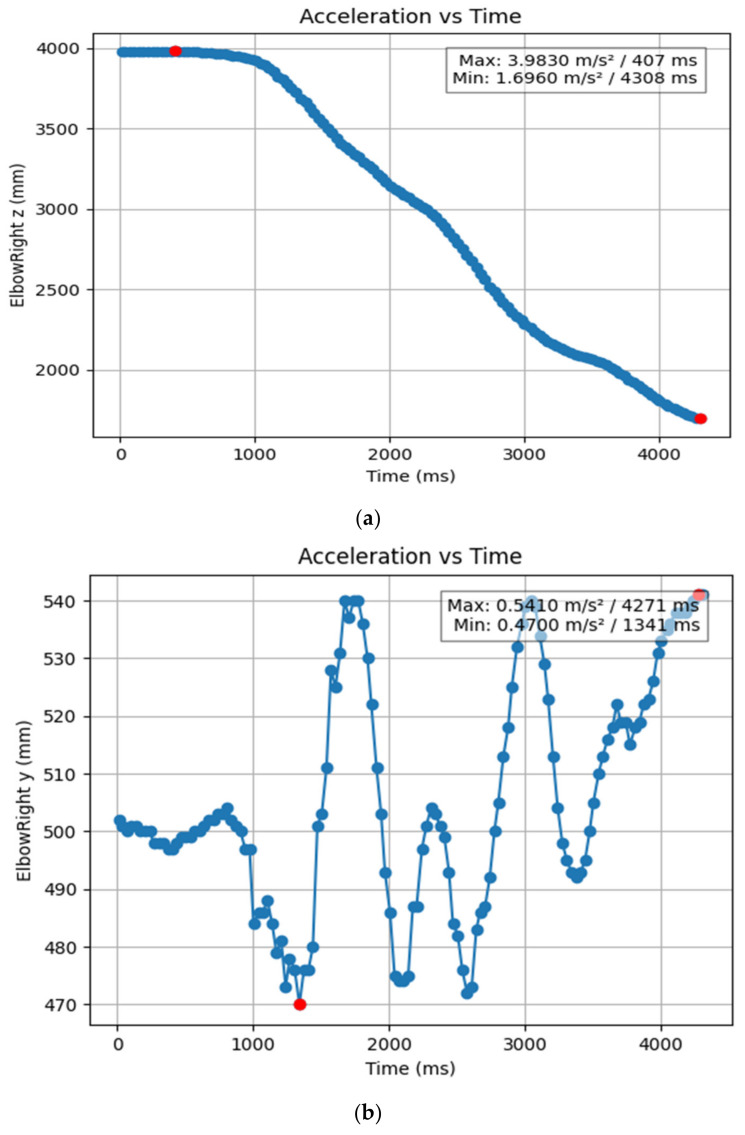

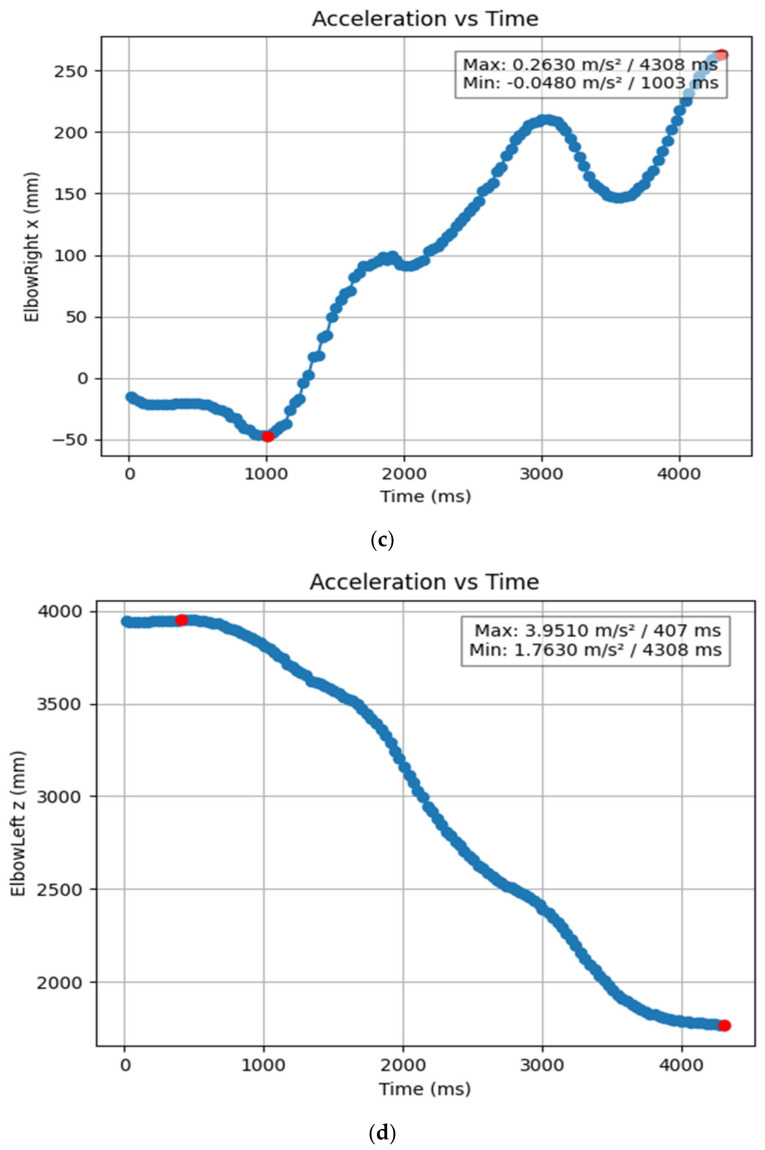

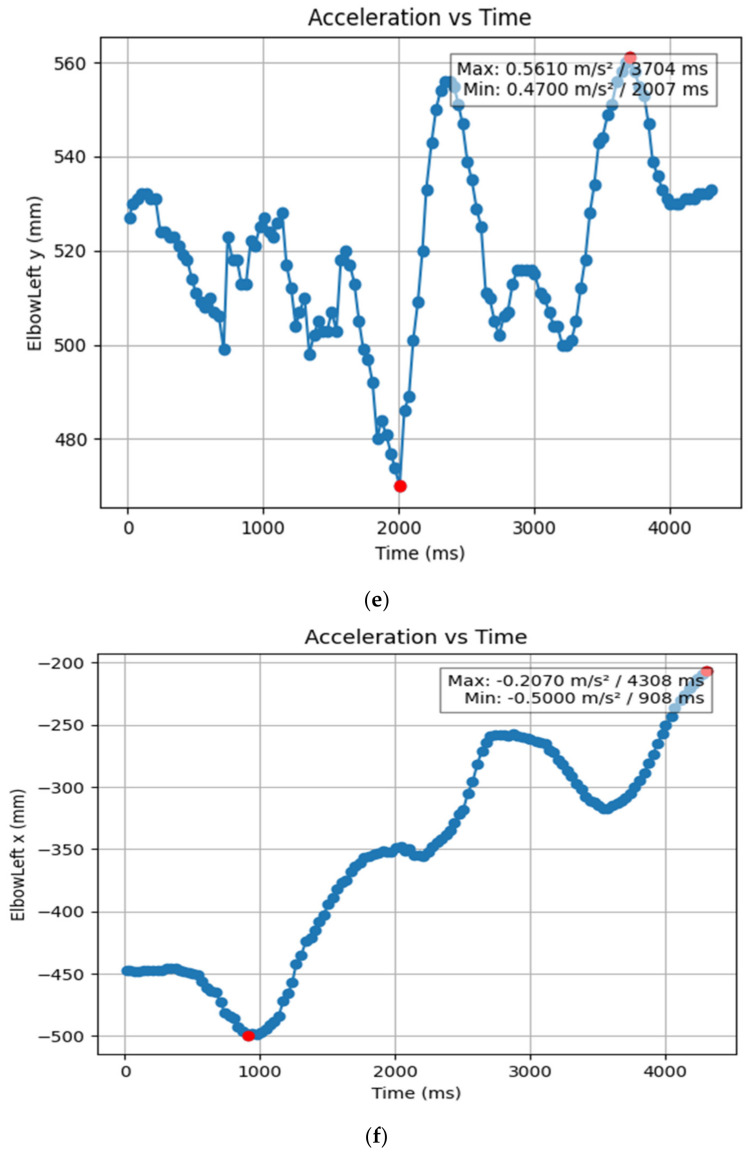
(**a**) Graph acceleration showing maximum and minimum points in the right elbow z direction angle position. (**b**) Graph acceleration showing maximum and minimum points in the right elbow y direction angle position. (**c**) Graph acceleration showing maximum and minimum points in the right hip x direction angle position. (**d**) Graph acceleration showing maximum and minimum points in the right hip x direction angle position. (**e**) Graph acceleration showing maximum and minimum points in the left elbow z direction angle position. (**f**) Graph acceleration showing maximum and minimum points in the left elbow x direction angle position.

**Figure 12:**
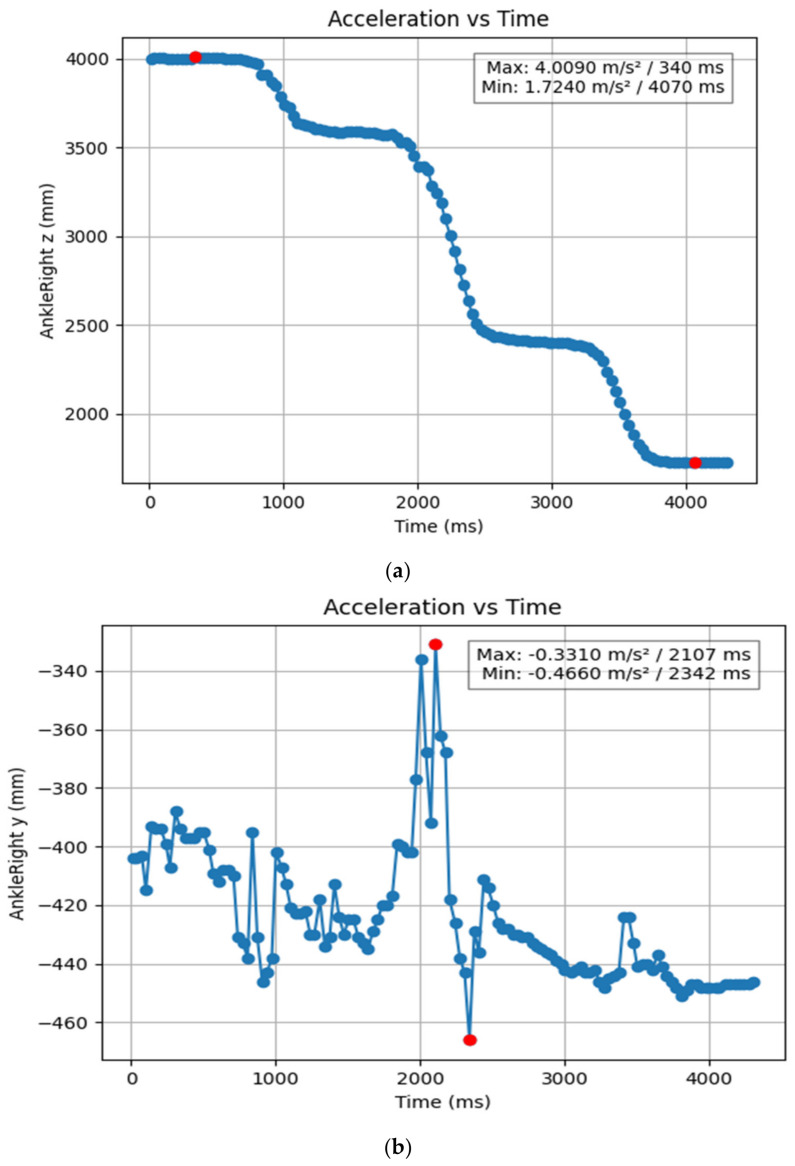

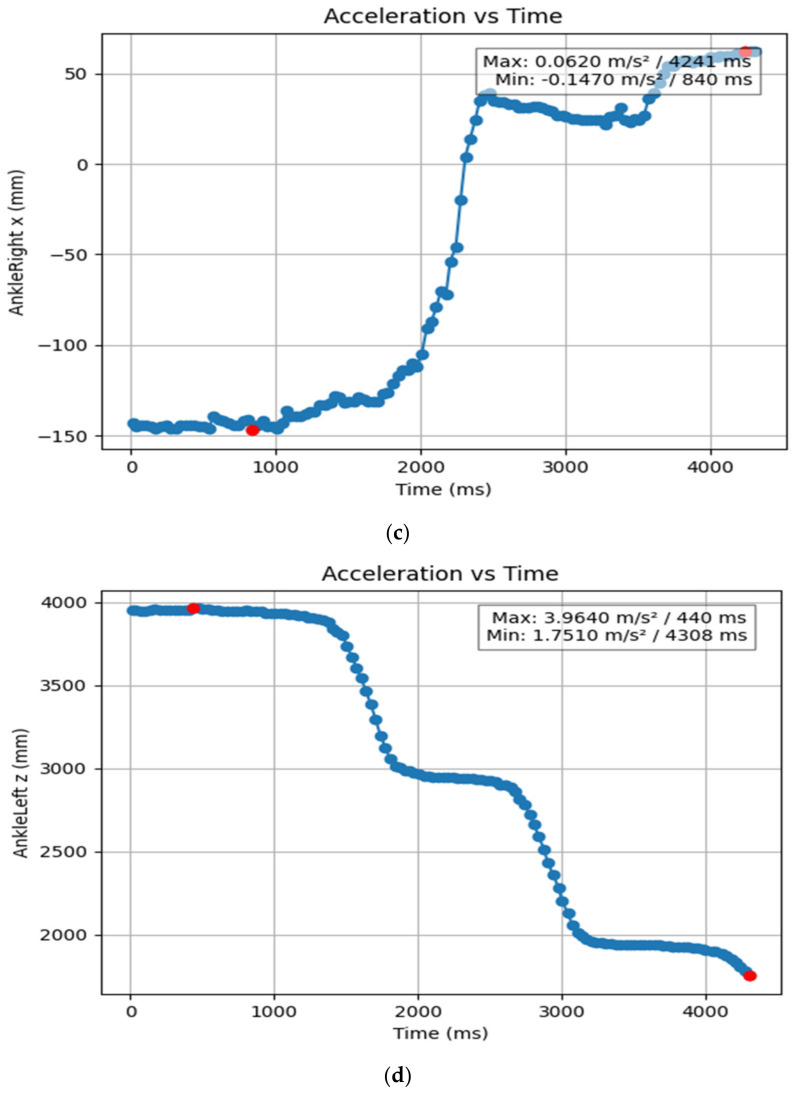

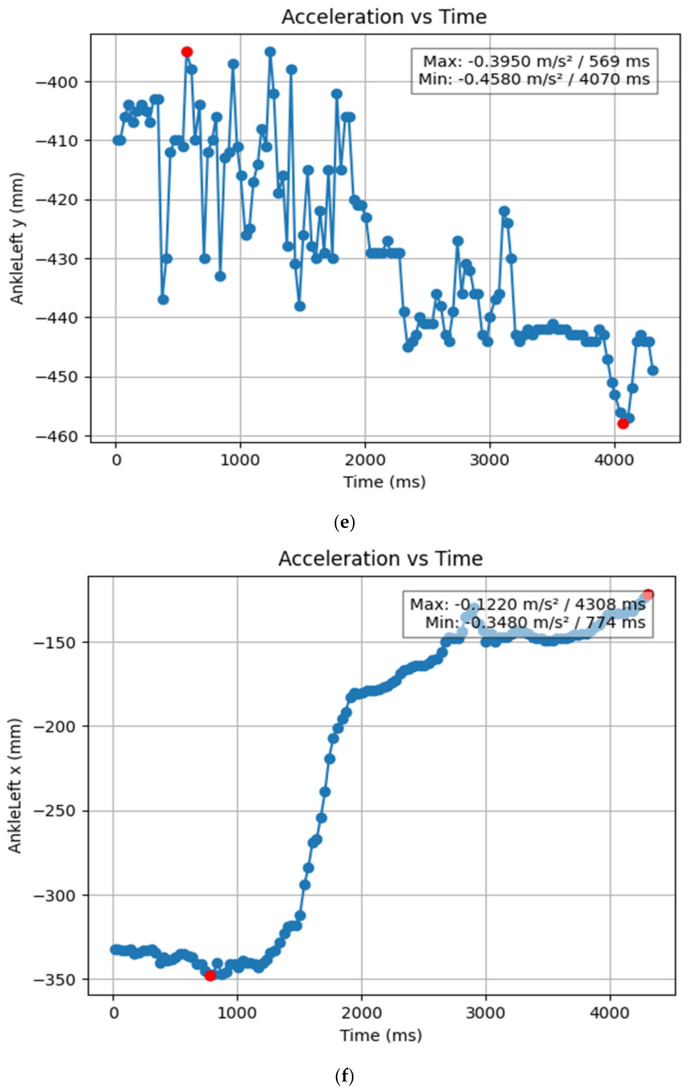
(**a**) Graph acceleration showing maximum and minimum points in the right ankle z direction angle position. (**b**) Graph acceleration showing maximum and minimum points in the right ankle y direction angle position. (**c**) Graph acceleration showing maximum and minimum points in the right ankle x direction angle position. (**d**) Graph acceleration showing maximum and minimum points in the left ankle z direction angle position. (**e**) Graph acceleration showing maximum and minimum points in the left ankle y direction angle position. (**f**) Graph acceleration showing maximum and minimum points in the left ankle x direction angle position.

**Figure 13:**
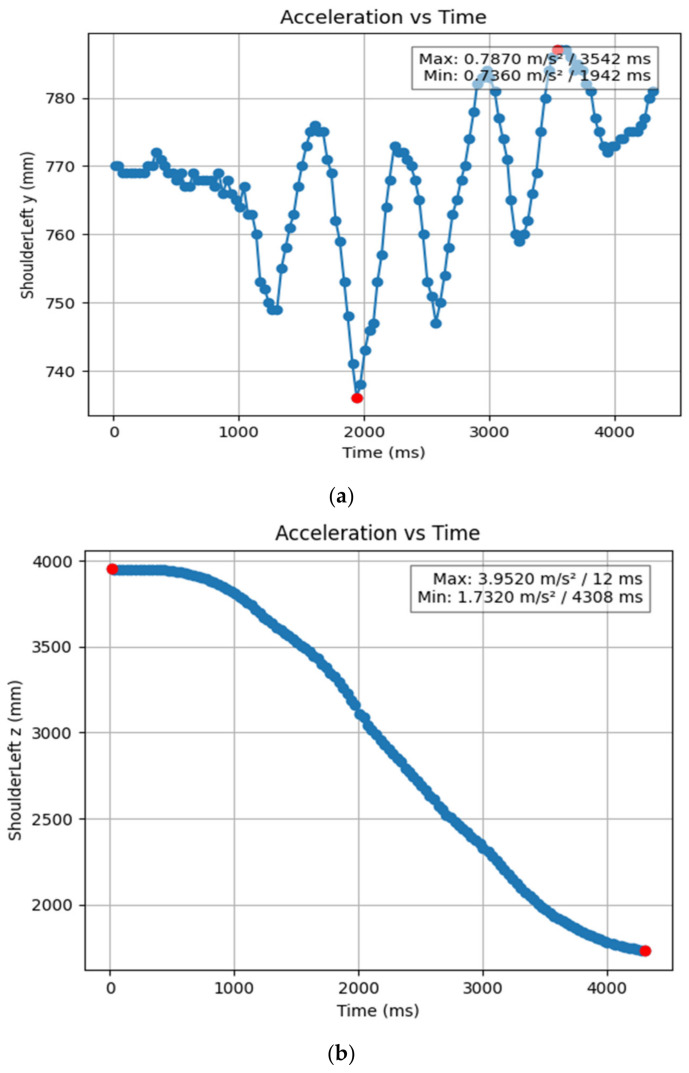
(**a**) Graph acceleration showing maximum and minimum points in the left shoulder y angle positions dancers. (**b**) Graph acceleration showing maximum and minimum points in the left shoulder z angle positions dancers.

**Figure 14:**
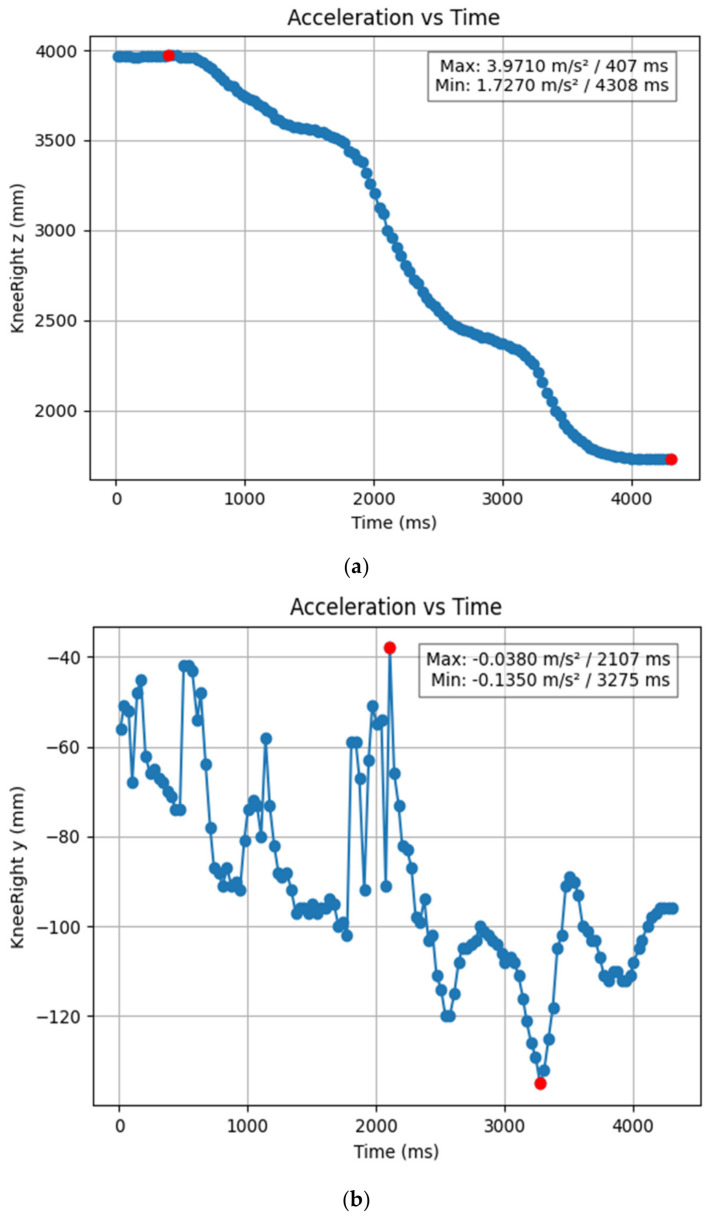

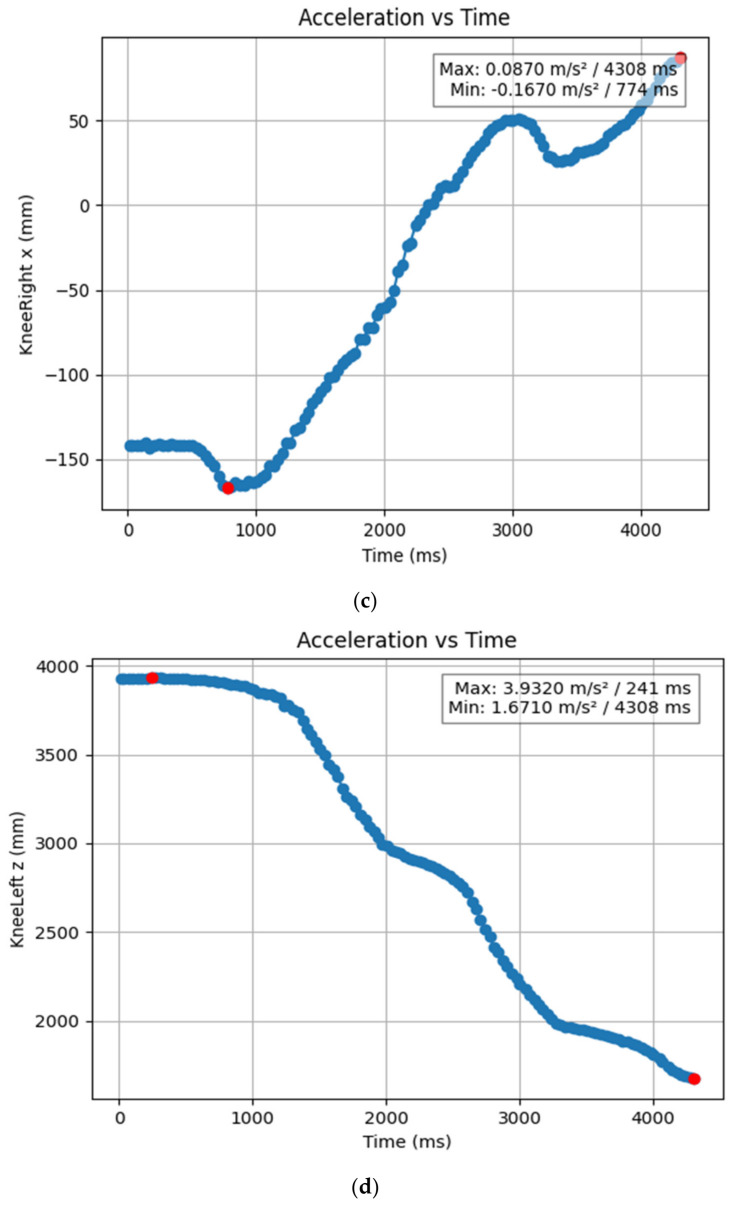

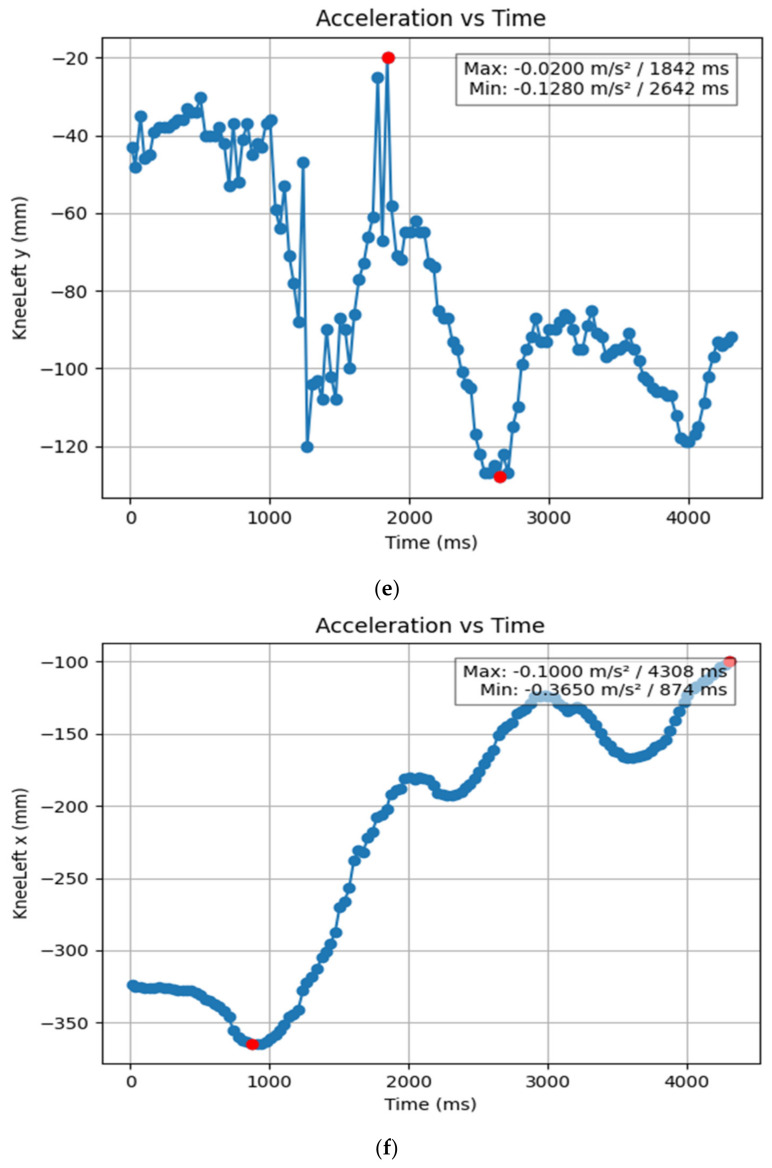
(**a**) Graph acceleration showing maximum and minimum points in the right knee z angle positions dancers. (**b**) Graph acceleration showing maximum and minimum points in the right. Knee y angle positions-dancers. (**c**) Graph acceleration showing maximum and minimum points in the right knee x angle positions—dancers. (**d**) Graph acceleration showing maximum and minimum points in the left knee z angle positions-dancers. (**e**) Graph acceleration showing maximum and minimum points in the left knee y angle positions-dancers. (**f**) Graph acceleration showing maximum and minimum points in the left knee y angle positions-dancers.

**Figure 15:**
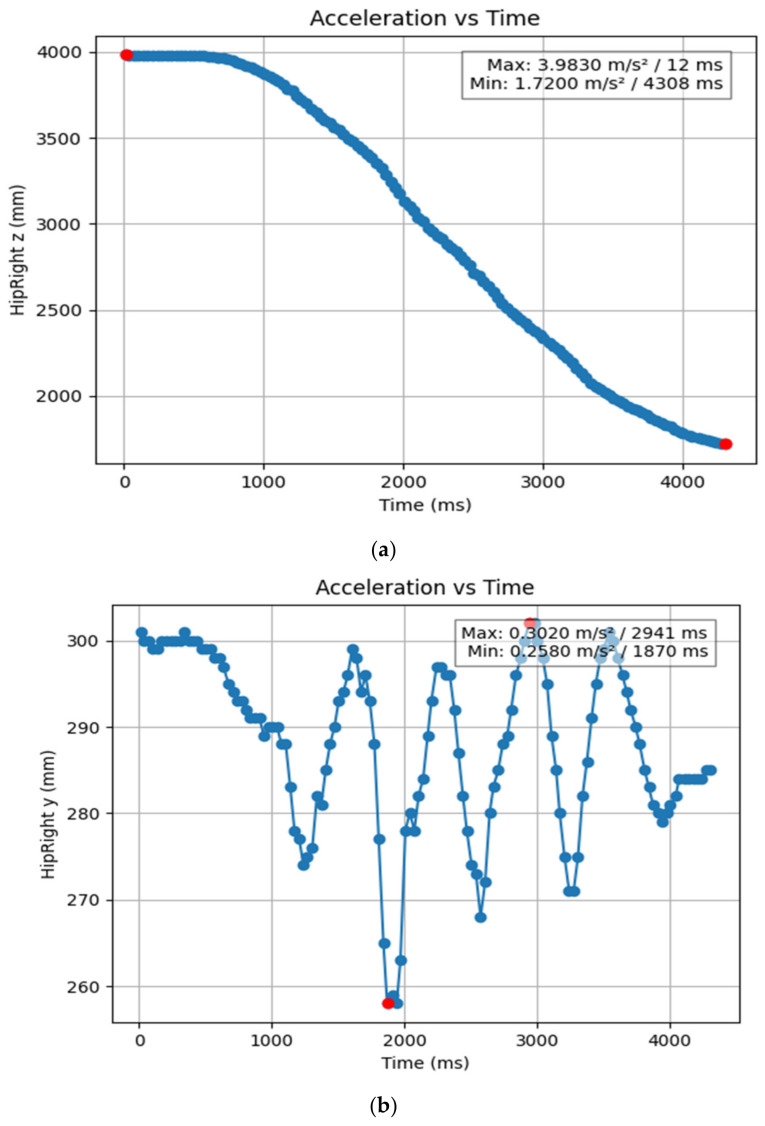

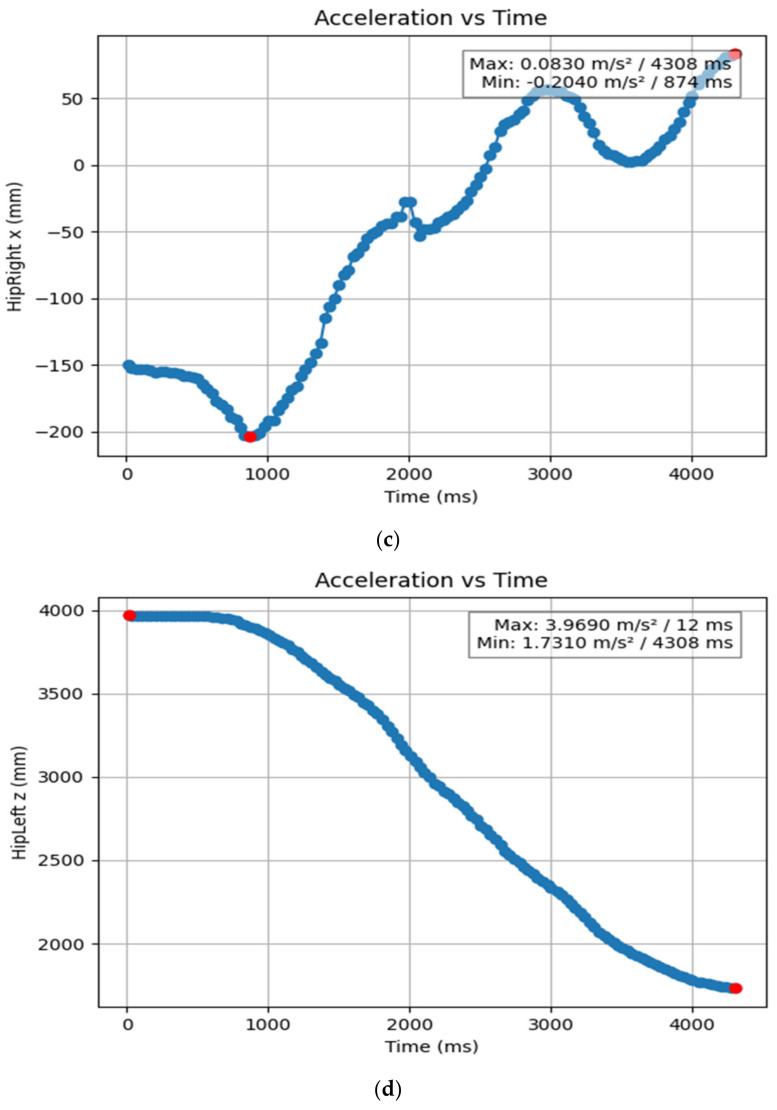

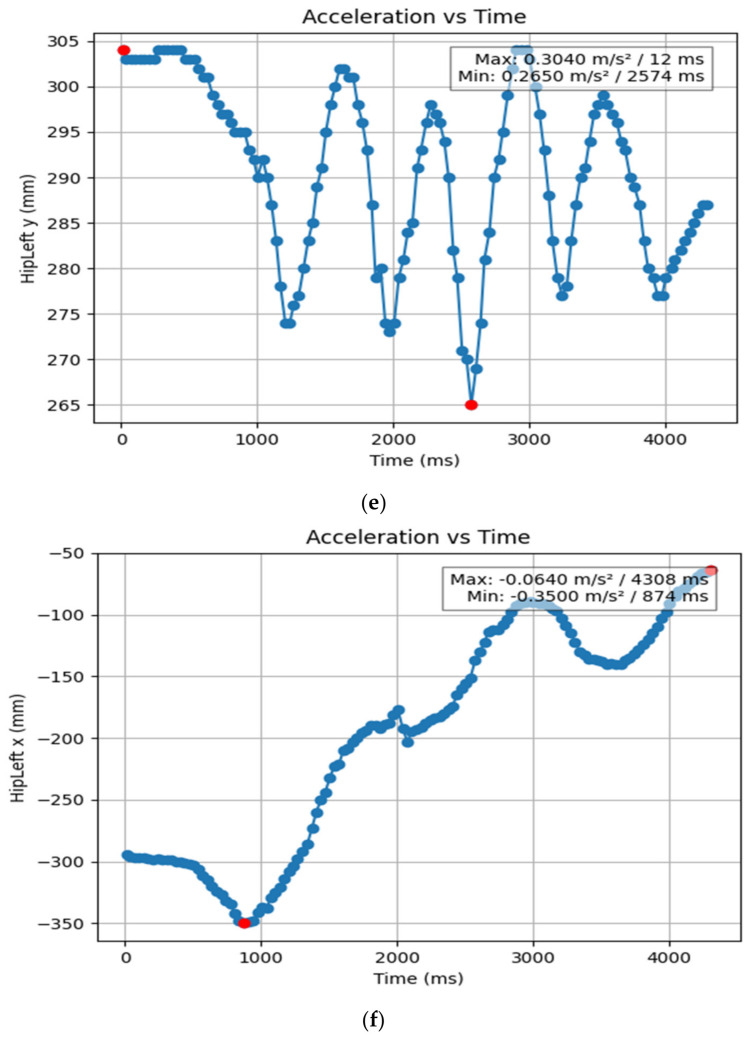
(**a**) Graph acceleration showing maximum and minimum points in the right hip z angle positions-dancers. (**b**) Graph acceleration showing maximum and minimum points in the right hip y angle positions-dancers. (**c**) Graph acceleration showing maximum and minimum points in the left hip x angle positions-dancers. (**d**) Graph acceleration showing maximum and minimum points in the left hip z angle positions-dancers. (**e**) Graph acceleration showing maximum and minimum points in the left hip y angle positions-dancers. (**f**) Graph acceleration showing maximum and minimum points in the left knee x angle positions-dancers.

**Figure 16:**
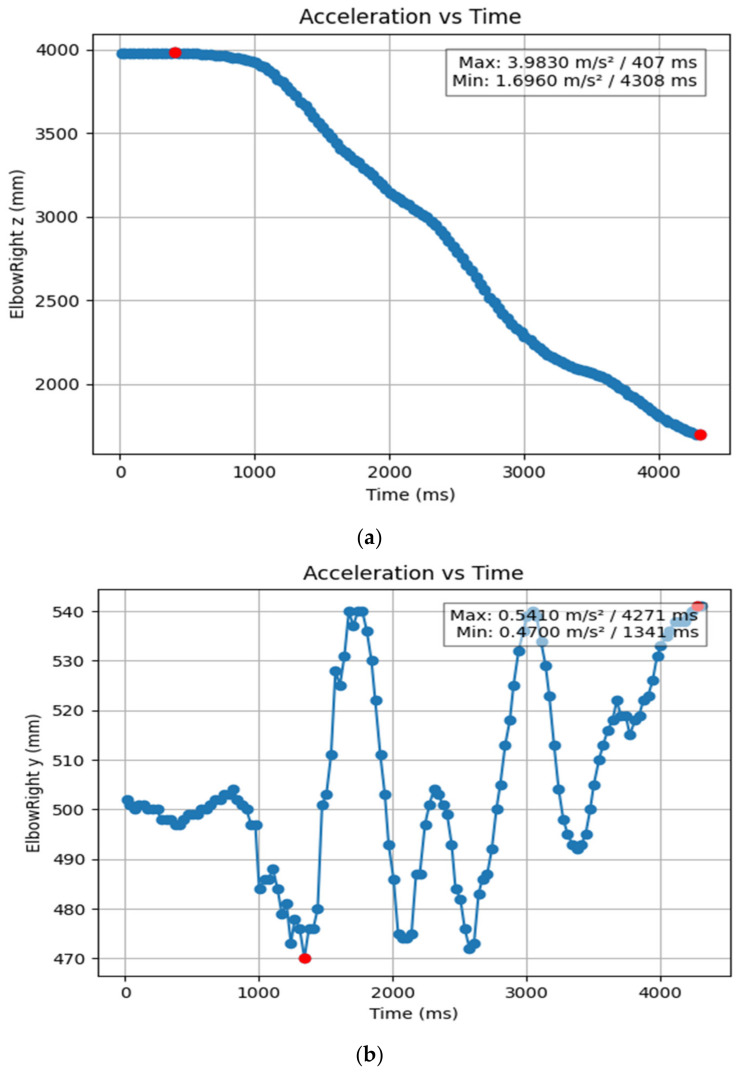

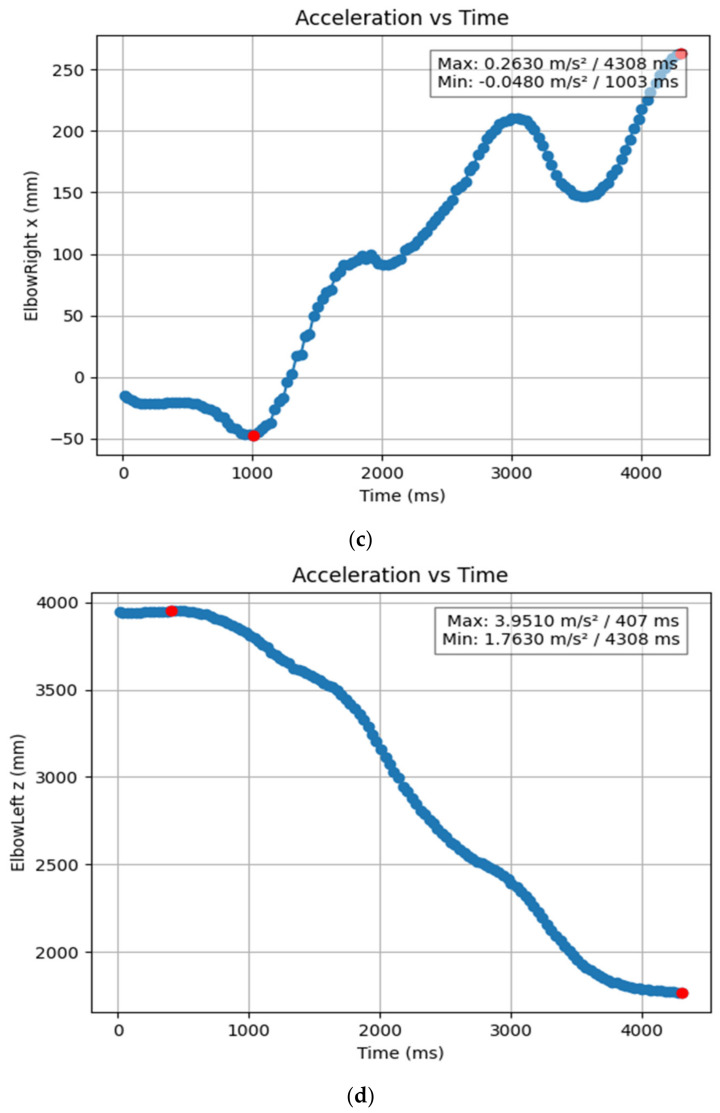

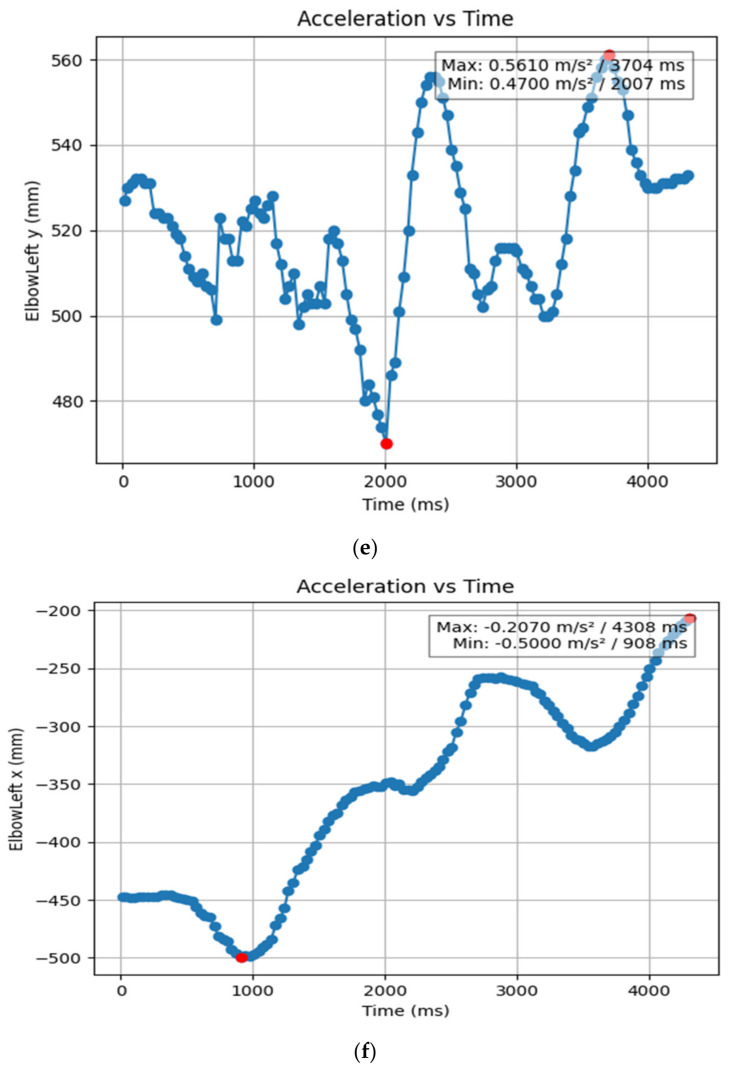
(**a**) Graph acceleration showing maximum and minimum points in the right elbow z angle positions-dancers. (**b**) Graph acceleration showing maximum and minimum points in the right elbow y angle positions-dancers. (**c**) Graph acceleration showing maximum and minimum points in the right elbow x angle positions-dancers. (**d**) Graph acceleration showing maximum and minimum points in the right elbow z angle positions-dancers. (**e**) Graph acceleration showing maximum and minimum points in the right elbow y angle positions-dancers. (**f**) Graph acceleration showing maximum and minimum points in the left elbow x angle positions-dancers.

**Figure 17:**
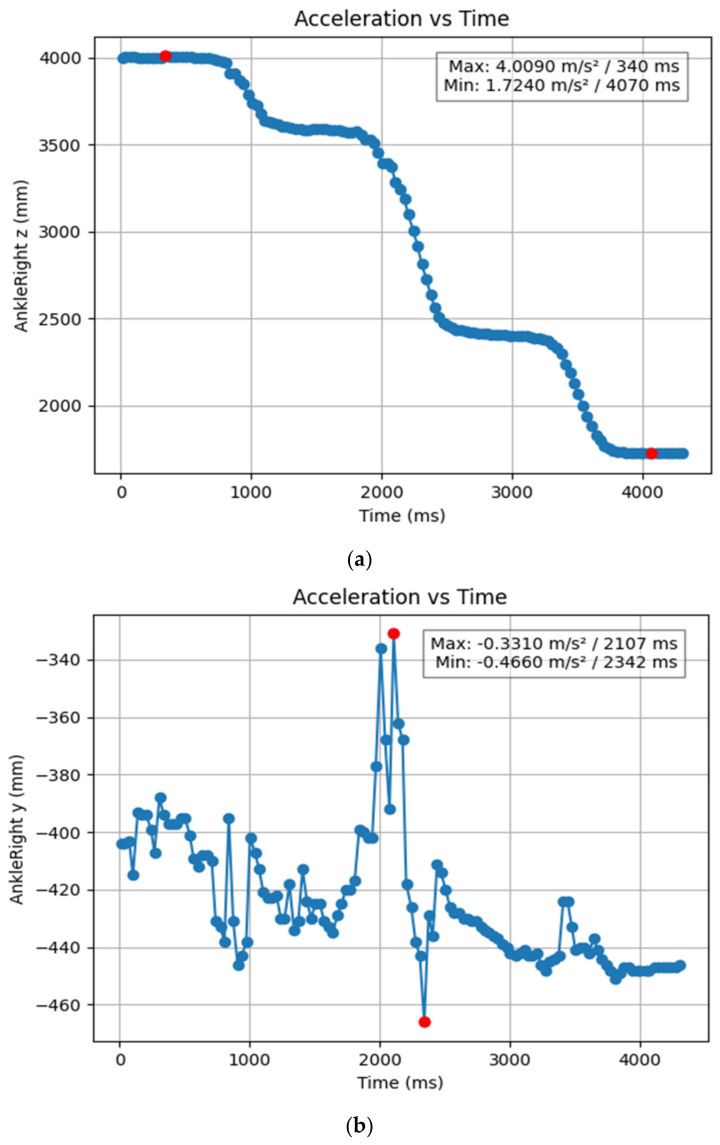

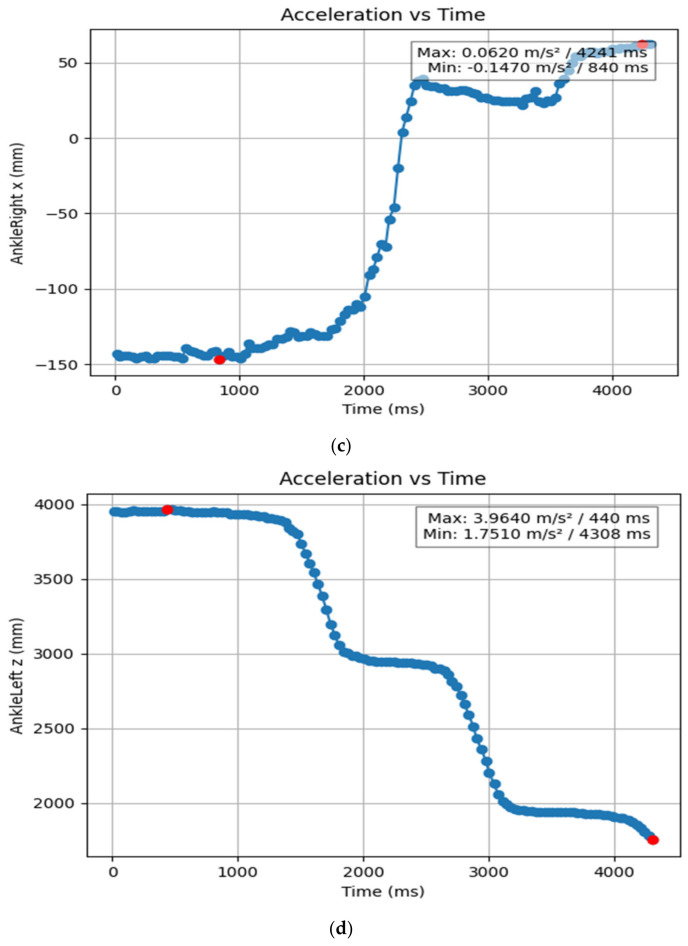

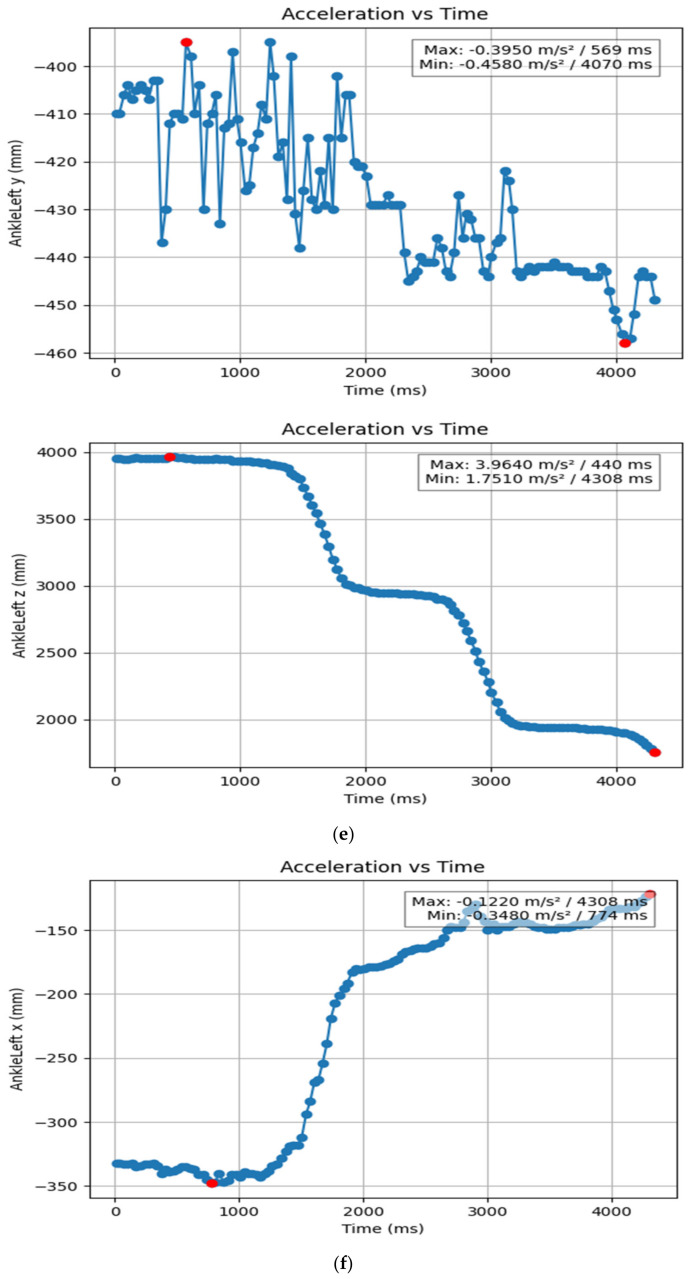
(**a**) Graph acceleration showing maximum and minimum points in the right ankle z angle positions-dancers. (**b**) Graph acceleration showing maximum and minimum points in the right ankle y angle positions-dancers. (**c**): Graph acceleration showing maximum and minimum points in the right ankle x angle positions- dancers. (**d**) Graph acceleration showing maximum and minimum points in the left ankle z angle positions-dancers. (**e**) Graph acceleration showing maximum and minimum points in the left ankle y angle positions-dancers. Graph acceleration showing maximum and minimum points in the left ankle z angle positions-dancers. (**f**) Graph acceleration showing maximum and minimum points in the left ankle x angle positions-dancers.

**Figure 18:**
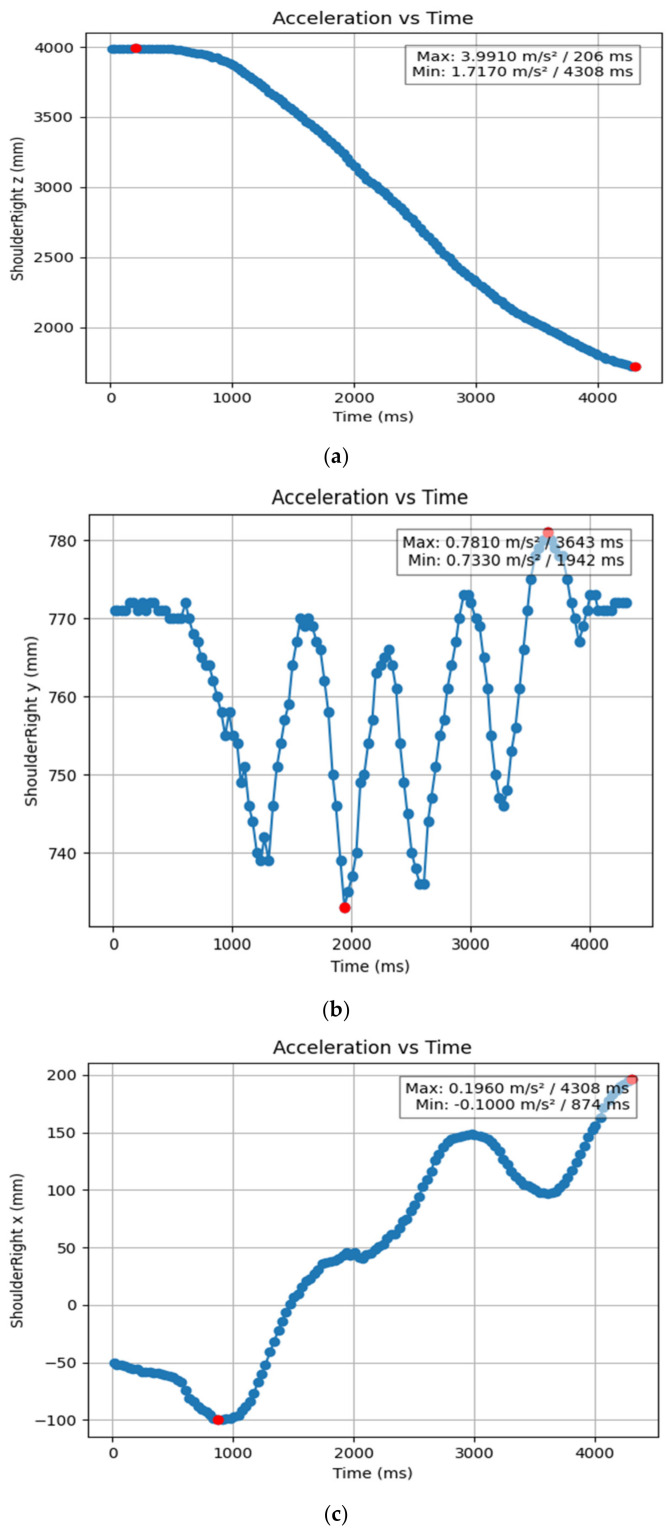
(**a**) Graph acceleration showing maximum and minimum points in the shoulder z angle positions-dancers. (**b**) Graph acceleration showing maximum and minimum points in the right shoulder y angle positions-dancers. (**c**) Graph acceleration showing maximum and minimum points in the right shoulder x angle positions-dancers.

Acceleration is a vector quantity that describes the rate of change of velocity over time, with both magnitude and direction (see Figure 8, Figure 9, Figure 10, Figure 11, Figure 12, Figure 13, Figure 14, Figure 15, Figure 16, Figure 17 and Figure 18). In such a table, positive values suggest acceleration in the positive direction of the respective axis, while negative values indicate acceleration in the opposite direction. The first step in analyzing these accelerations is to identify the acceleration values for each body part and axis (*x*, *y*, *z*). These values reveal how quickly the speed of the body part is changing in the respective direction. Positive values reflect an increase in velocity in the direction of the axis, while negative values indicate deceleration or acceleration in the opposite direction. The directional analysis of the acceleration values provides deeper insights into the type of movement. Acceleration along the *x*-axis refers to left-right lateral movements or swaying, *y*-axis acceleration is related to vertical movements such as jumping or crouching, and *z*-axis acceleration signifies forward or backward stepping or depth-related movements. A comparative analysis of acceleration across different body parts allows us to infer which body parts are accelerating faster, slowing down, or changing direction. For instance, higher positive acceleration in the right_ ankle compared to the left ankle might indicate a motion such as kicking where the foot moves faster than the hip. Movement coordination can also be evaluated by analyzing the timing and coordination of accelerations across various body parts during complex motions. For example, in a jumping motion, you would expect to see positive acceleration in the *y*-axis of the legs first, followed by the rest of the body. Additionally, noteworthy patterns or abnormalities in acceleration values can signal important insights. Sudden changes in acceleration or discrepancies between symmetric body parts (e.g., left and right ankles) could point to potential inefficiencies, deliberate movement nuances, or even injury risks. Taking a few specific examples from such data, left shoulder *x*-axis acceleration shows −152 mm, indicating deceleration or movement towards the left (assuming positive x is rightward); right ankle *y*-axis acceleration is −286 mm, which suggests a downward movement, likely from a jump or step; and left knee *z*-axis acceleration at 4202 mm shows a very high positive value, suggesting rapid forward movement or an extension of the left knee. These observations provide insights into the nature of the movement being captured, whether it involves running, jumping, or throwing, based on how different body parts accelerate relative to each other during the movement (Table 4 and Table 5).

### 3.2. Acceleration Graphs with Regression Equation Lines

**Figure 19:**
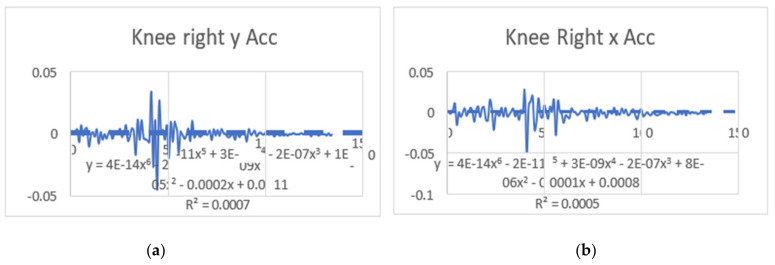
(**a**) Right knee with regression equation line. (**b**) Left knee with regression equation line.

**Figure 20:**
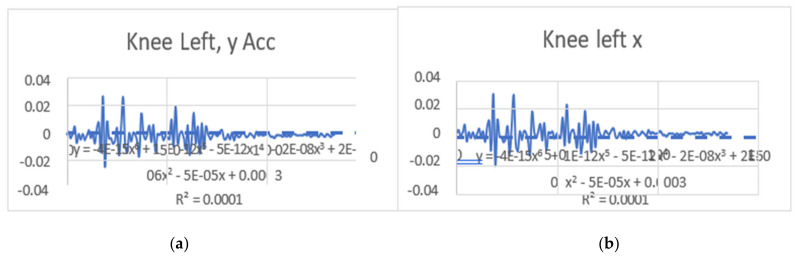
(**a**) left knee y direct with regression equation line. (**b**) Left knee x with regression equation line.

**Figure 21:**
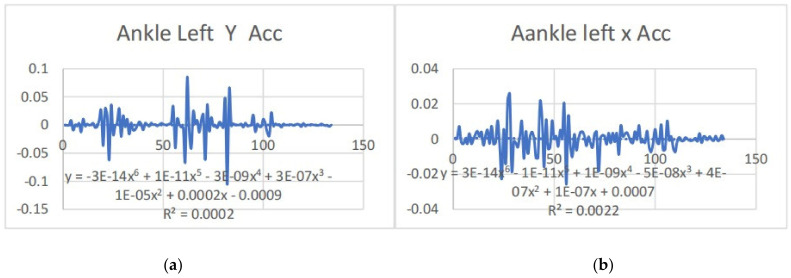
(**a**) left ankle y with regression equation line. (**b**) Left ankle x with regression equation line.

**Figure 22:**
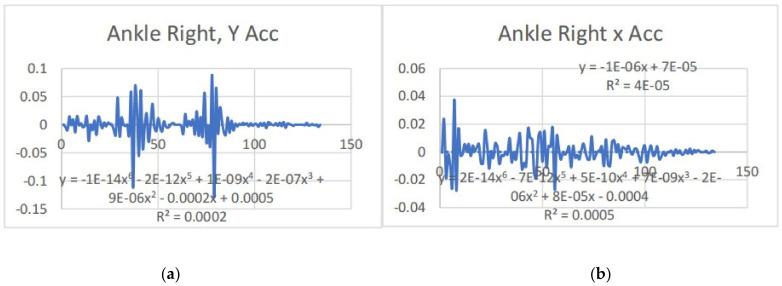
(**a**) Right ankle y with regression equation line. (**b**) Right ankle x with regression equationc.

**Figure 23:**
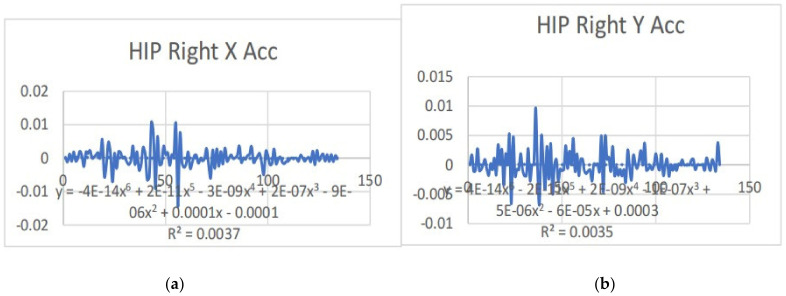
(**a**) Right Hip with regression equation line. (**b**) Right Hip with regression equation line.

**Figure 24:**
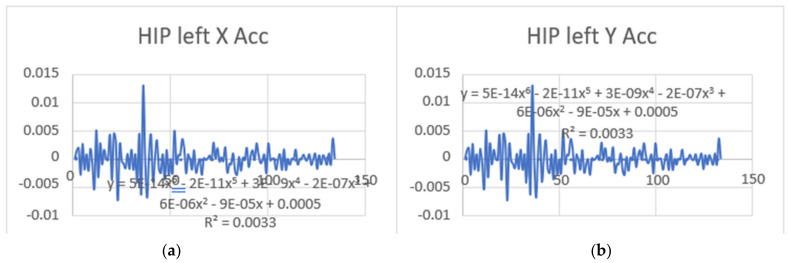
(**a**) Left Hip with regression equation line. (**b**) Left Hip with regression equation line.

**Figure 25:**
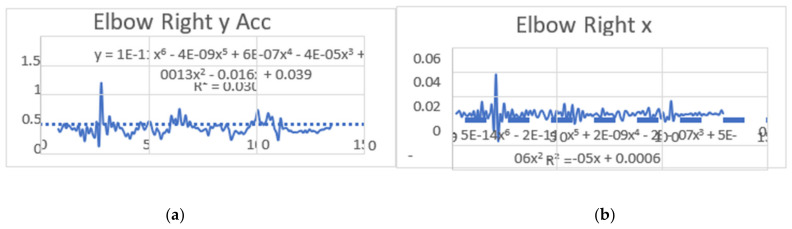
(**a**) Right elbow with regression equation line. (**b**) Elbow elbow with regression equation line.

**Figure 26:**
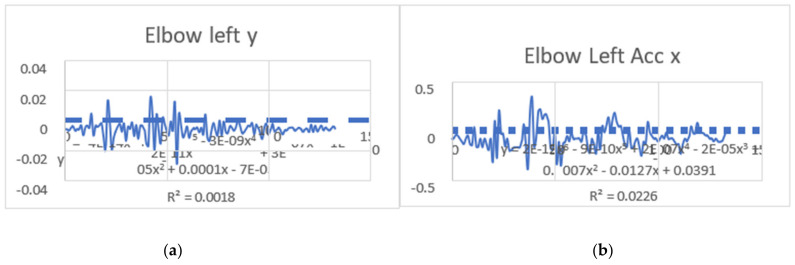
(**a**) left elbow with regression equation line. (**b**) left elbow with regression equation line.

**Figure 27:**
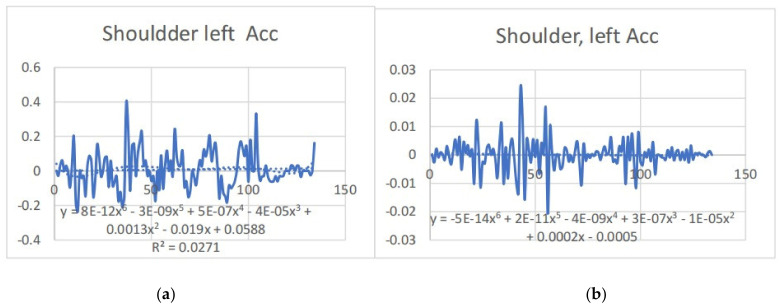
(**a**) left shoulder y with regression equation line. (**b**) left shoulder x with regression equation line.

**Figure 28:**
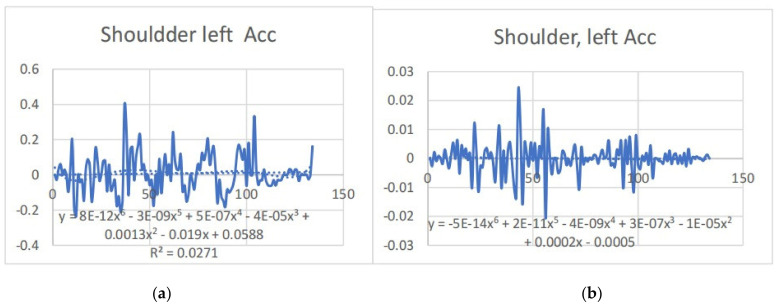
(**a**) Right shoulder y with regression equation line. (**b**) Right shoulder x with regression equation line.

The gait analysis and acceleration data delve into the complexities of movement patterns of professional dancers compared to non-dancers, focusing on how acceleration, a measure of how quickly the velocity of a body part changes, plays a crucial role in understanding biomechanics (see Figure 19, Figure 20, Figure 21, Figure 22, Figure 23, Figure 24, Figure 25, Figure 26, Figure 27 and Figure 28). The research meticulously examines the positioning and movement of various body parts (knees, ankles, hips, elbows, shoulders) in two dimensions (x and y), capturing both angular velocities and accelerations during walking and dance routines. These data are summarized in detailed tables and graphs above, complemented by polynomial regression equations and their corresponding R-squared values to model the dynamic relationship between velocity and acceleration across different joints.

Acceleration is revealed to be far from constant; it varies significantly with the activity, showing marked changes during dance compared to walking. This is attributed to the diverse demands these activities place on the body’s biomechanics. The acceleration data, systematically organized and analyzed, highlights how acceleration changes with velocity but also suggests that these changes are not always linear or proportional. Different joints respond differently to the same overall body movement due to the distinct forces acting on them, leading to unique acceleration and deceleration patterns that are crucial for understanding the dynamics of movement (Table 6).

The interplay between velocity and acceleration is foundational in assessing kinetic and potential energy shifts within the body. An increase in velocity leads to a rise in kinetic energy, the energy a body possesses due to motion. Meanwhile, potential energy stored due to position or posture changes, particularly during dance movements, is directly influenced by how acceleration and deceleration are managed across different body parts. Through this analysis, the study provides deep insights into the energy dynamics of human movement, underlining the importance of acceleration in evaluating biomechanical efficiency and energy expenditure.

Understanding these intricate relationships offers valuable implications for performance enhancement, injury prevention, and rehabilitation strategies. It underscores the necessity for targeted training and interventions that consider the biomechanical principles of acceleration and velocity changes. By accounting for how different body parts uniquely respond to the demands of various activities, professionals can develop more effective approaches to improving dance performance and general movement health, benefiting not just the dance community but also individuals seeking to optimize their physical capabilities.

## 4. Discussion

The study’s results show notable variations in angular velocity and acceleration between professional dancers and non-dancers, offering information on how intense training affects joint control and movement effectiveness. Narrower ranges of angular velocities and accelerations were consistent with more accurate and regulated movement patterns in dancers than in non-dancers. The angular velocity of the right knee, for instance, varied between −3.88 and 2.61 rad/s in non-dancers and a significantly narrower range of −0.35 to 0.54 rad/s in dancers, demonstrating improved neuromuscular coordination. The acceleration data also revealed that dancers maintained more stable deceleration, such as a shoulder deceleration of −0.1 mm/s^2^, which contrasts with the broader, less controlled acceleration patterns observed in non-dancers, like the left elbow’s acceleration of 2.997 mm/s^2^. These results align with previous studies that demonstrate how dance training enhances motor control and proprioception, allowing for more efficient biomechanical performance [25]. Prior research has similarly found that trained individuals, such as dancers, can optimize their movements, minimizing energy wastage and reducing the risk of injury [26]. The idea that inefficient and maybe harmful movement results from a lack of fine motor control is supported by the increased variety in joint movement patterns among non-dancers. As reported in the literature, this variability was observed in both a greater range of angular velocities and irregular acceleration and deceleration patterns, indicating that persons who are not trained may exert more energy and be more susceptible to strain during physical exercise. These results have significant ramifications for the dance community as well as those working in physical rehabilitation. For dancers, the results underscore the importance of training in developing efficient movement patterns that enhance performance while reducing the risk of injury [27]. For non-dancers, the study suggests that targeted training programs focusing on improving kinematic control could help reduce the variability in movement patterns and, consequently, the risk of injury. Rehabilitation programs could incorporate these findings to develop interventions that improve motor control and movement efficiency, particularly for individuals recovering from injuries or those with mobility challenges. A comparative investigation of dancers and non-dancers is one of the study’s strengths, as is the use of high-resolution motion capture technology, which made it possible to measure angular velocity and acceleration precisely. However, this research has limitations. With only four professional dancers and six non-dancers, the sample size was tiny, which might have limited how broadly the results can be applied. Furthermore, because ballet dancers were the exclusive focus of the study, the findings might not apply to other dancers or athletes. Future research could expand on this work by including a larger and more diverse sample, including participants from different training backgrounds, or by exploring other forms of movement such as athletic performance or contemporary dance. Investigating how different types of training affect joint control and movement efficiency over time would also provide valuable insights. Future research could also focus on the longitudinal effects of targeted training interventions for non-dancers, examining how quickly and to what extent their movement patterns can be improved. Integrating advanced technologies such as 3D motion capture and machine learning models could further enhance the analysis of muscle coordination and kinematic efficiency, leading to improved training protocols, injury prevention strategies, and performance enhancement techniques for dancers, athletes, and the general population.

## 5. Conclusions

This research has effectively used cutting-edge motion capture technology to identify important biomechanical differences in angular velocities and accelerations between professional ballet dancers and non-dancers. Clear insights into how ballet training improves biomechanical efficiency and movement control are offered by the findings. The main findings highlight that dancers exhibit significantly more controlled angular velocities, particularly in the right knee, where non-dancers displayed a broader range (−3.88 to 2.61 rad/s), compared to the narrower and more refined range of dancers (−0.35 to 0.54 rad/s). This distinction emphasizes the superior kinematic control that dancers develop through rigorous training. Similarly, acceleration data revealed that dancers manage accelerative forces with greater precision, as evidenced by a maximum left shoulder acceleration of 3.952 mm/s^2^ and a minimum of −0.1 mm/s^2^ in the right shoulder. Non-dancers, however, exhibited a wider and less controlled range of accelerations, such as a maximum of 2.997 mm/s^2^ in the left elbow.

These findings have practical implications for developing more effective training programs that enhance performance while reducing the risk of injury. Understanding the clear distinctions in movement dynamics between trained and untrained individuals provides a scientific foundation for refining training regimens in the fields of dance, sports, and rehabilitation.

In conclusion, by providing insightful information about movement efficiency and control, this study advances our knowledge of how training affects biomechanical characteristics. The study shows the value of specialized training in attaining biomechanical excellence by recording both angular velocities and accelerations. This helps not just dancers, but also other groups involved in movement health and performance optimization.

## Figures and Tables

**Table 1:** Angular velocity of joint F-plane (m/s) for non-dancers.

Non-Dancer	Gait	Angular Velocity_Min	Angular_Velocity Max	Time_Min	Time_Max
	Left Shoulder	−2.13	1.55	3249.65	3219.91
	Right Shoulder	−0.47	0.71	3223.63	3253.37
	Left Elbow	−2.54	2.66	3625.05	3651.07
	Right Elbow	−1.26	1	3249.65	3219.91
	Left Hip	−0.49	0.25	3249.65	2803.63
	Right Hip	−0.85	0.83	3651.07	3249.65
	Left Knee	−1.26	1.65	3625.05	3651.07
	Right Knee	−3.88	2.61	3249.65	3219.91
	Left Ankle	−1.96	1.75	3227.35	3260.8
	Right Ankle	−1.31	1.49	4740.11	2870.53

**Table 2:** Angular velocity of joint F-plane (m/s) for dancers.

Dancer	Gait	Angular Velocity_min	Angular Velocity_max	Time_min	Tim_max
	Left Shoulder	−0.09	0.08	1178.28	3148.22
	Right Sholder	−0.09	0.07	1246.44	784.73
	Left Elbow	−0.28	0.27	3146.03	1275.02
	Right Elbow	−0.27	0.22	978.21	1873.04
	Left Hip	−0.16	0.09	1437.71	1508.07
	Right Hip	−0.18	0.14	1376.15	1417.93
	Left Knee	−0.29	0.47	1903.82	971.61
	Right Knee	−0.35	0.54	1395.94	1631.19
	Left Ankle	−1.48	1.34	613.24	571.47
	Right Ankle	−1.39	1.66	1580.62	1538.85

**Table 3:** Angular positions with regression equation lines.

Left	Poly-Regression Equation	Slope	Right	Poly-Regression Equation	Slope
Shoulder x	y = 1E-09 × ^6^ − 1E-06 × ^5^ + 0.0003x^4^ − 0.0352x^3^ + 2.1241x^2^ − 47.302x + 193.92 R^2^ = 0.6182	y = 0.6507x − 3.006 R^2^ = 0.1171	shoulder x	y = 1E-09x^6^ − 9E-07x^5^ + 0.0003x^4^ − 0.0333x^3^ + 1.9364x^2^ − 40.072x + 460.92	y = 0.7381x + 337.02 R^2^ = 0.1457
Shoulder y	y = 1E-10x^6^ − 9E-08x^5^ + 2E-05x^4^ − 0.0021x^3^ + 0.0979x^2^ − 0.099x + 250.78 R^2^ = 0.3963	y = −0.0319x + 320.66 R^2^ = 0.0012	shoulder y	y = 1E-09x^6^ − 9E-07x^5^ + 0.0003x^4^ − 0.0333x^3^ + 1.9364x^2^ − 40.072x + 460.92 R^2^ = 0.5961	y = 0.7381x + 337.02 R^2^ = 0.1457
Shoulder z	y = −4E-10x^6^ + 3E-07x^5^ − 0.0001x^4^ + 0.0179x^3^ − 1.4477x^2^ + 50.546x + 2218.7 R^2^ = 0.3584	y = 1.3928x + 2620.9 R^2^ = 0.1822	shoulder y	y = −1E-09x^6^ + 8E-07x^5^ − 0.0002x^4^ + 0.0342x^3^ − 2.3942x^2^ + 70.852x + 2094.2	y = 1.4539x + 2578.4 R^2^ = 0.2022
Elbow x	y = 7E-10x^6^ − 5E-07x^5^ + 0.0002x^4^ − 0.0208x^3^ + 1.3123x^2^ − 31.648x + 63.285 R^2^ = 0.6068	y = 0.5824x − 139.74 R^2^ = 0.1299	elbow x	y = 1E-09x^6^ − 1E-06x^5^ + 0.0003x^4^ − 0.0323x^3^ + 1.8226x^2^ − 34.944x + 482.63 R^2^ = 0.709	y = 0.9853x + 451.97 R^2^ = 0.2351
Elbow y	y = 9E-10x^6^ − 6E-07x^5^ + 0.0002x^4^ − 0.0215x^3^ + 1.2826x^2^ − 26.74x + 193.95 R^2^ = 0.7059	y = − 0.1198x + 229.51 R^2^ = 0.0033	elbow y	y = 6E-10x^6^ − 4E-07x^5^ + 9E-05x^4^ − 0.0094x^3^ + 0.4989x^2^ − 8.1204x + 77.445 R^2^ = 0.7335	y = 0.194x + 168.92 R^2^ = 0.0102
Elbow z	y = 6E-10x^6^ − 4E-07x^5^ + 8E-05x^4^ − 0.0065x^3^ − 0.0373x^2^ + 20.295x + 2314.6 R^2^ = 0.3714	y = 1.3233x + 2598.5 R^2^ = 0.137	elbow z	y = −1E-09x^6^ + 1E-06x^5^ − 0.0003x^4^ + 0.0447x^3^ − 3.0596x^2^ + 86.648x + 1960.6 R^2^ = 0.4629	y = 1.5943x + 2518.2 R^2^ = 0.2049
Hip x	y = 1E-09x^6^ − 1E-06x^5^ + 0.0003x^4^ − 0.0355x^3^ + 2.0766x^2^ − 44.289x + 265.59 R^2^ = 0.6356	y = 0.6655x + 93.149 R^2^ = 0.1287	hip x	y = 1E-09x^6^ − 1E-06x^5^ + 0.0003x^4^ − 0.0332x^3^ + 1.9213x^2^ − 39.799x + 386.81 R^2^ = 0.6154	y = 0.6643x + 252.81 R^2^ = 0.131
Hip y	y = 3E-10x^6^ − 2E-07x^5^ + 6E-05x^4^ − 0.0072x^3^ + 0.4247x^2^ − 9.9466x − 130.31 R^2^ = 0.414	y = − 0.0188x − 159.83 R^2^ = 0.0006	hip y	y = 3E-10x^6^ − 2E-07x^5^ + 5E-05x^4^ − 0.0063x^3^ + 0.3608x^2^ − 8.2405x − 135.68 R^2^ = 0.3956	y = −0.0023x − 160.38 R^2^ = 9E-06
Hip z	y = −7E-10x^6^ + 5E-07x^5^ − 0.0002x^4^ + 0.024x^3^ − 1.7672x^2^ + 56.652x + 2122.6 R^2^ = 0.3477	y = 1.3895x + 2566.4 R^2^ = 0.202	hip x	y = −9E-10x^6^ + 7E-07x^5^ − 0.0002x^4^ + 0.0286x^3^ − 2.0299x^2^ + 62.143x + 2086.5 R^2^ = 0.3598	y = 1.3919x + 2551.6 R^2^ = 0.2049
Knee x	y = 8E-19x^6^ − 2E-14x^5^ + 2E-10x^4^ − 7E-07x^3^ + 0.0014x^2^ − 0.9599x + 239.35 R^2^ = 0.7057	y = 0.0264x + 54.066 R^2^ = 0.2316	knee x	y = 1E-18x^6^ − 2E-14x^5^ + 2E-10x^4^ − 8E-07x^3^ + 0.0016x^2^ − 1.0698x + 349.77 R^2^ = 0.6636	y = 0.0171x + 286.8 R^2^ = 0.1211
Knee y	y = 4E-19x^6^ − 8E-15x^5^ + 7E-11x^4^ − 3E-07x^3^ + 0.0006x^2^ − 0.3986x − 487.24 R^2^ = 0.6997	y = − 0.0064x − 467.95 R^2^ = 0.0513	knee y	y = −2E-20x^6^ + 6E-16x^5^ − 6E-12x^4^ + 2E-08x^3^ − 9E-06x^2^ + 0.0137x − 558.69 R^2^ = 0.774	y = −0.0013x − 488.99 R^2^ = 0.0021
Knee z	y = −3E-19x^6^ + 7E-15x^5^ − 8E-11x^4^ + 4E-07x^3^ − 0.0008x^2^ + 0.58x + 2556.9 R^2^ = 0.8782	y = 0.0422x + 2487.8 R^2^ = 0.4272	knee z	y = 5E-19x^6^ − 1E-14x^5^ + 9E-11x^4^ − 3E-07x^3^ + 0.0004x^2^ − 0.253x + 2678.1 R^2^ = 0.8788	y = 0.0336x + 2517.1 R^2^ = 0.3122
Ankle x	y = 8E-19x^6^ − 2E-14x^5^ + 2E-10x^4^ − 8E-07x^3^ + 0.0015x^2^ − 1.0479x + 249.71 R^2^ = 0.5062	y = 0.0242x + 75.242 R^2^ = 0.151	ankle x	y = 9E-19x^6^ − 2E-14x^5^ + 2E-10x^4^ − 7E-07x^3^ + 0.0013x^2^ − 0.8189x + 317.65 R^2^ = 0.5931	y = 0.02x + 268.69 R^2^ = 0.1924
Ankle y	y = 3E-19x^6^ − 8E-15x^5^ + 7E-11x^4^ − 3E-07x^3^ + 0.0006x^2^ − 0.4268x − 772.93 R^2^ = 0.6686	y = − 0.0133x − 774.4 R^2^ = 0.2372	ankle y	y = −2E-19x^6^ + 5E-15x^5^ − 4E-11x^4^ + 2E-07x^3^ − 0.0003x^2^ + 0.182x − 863.2 R^2^ = 0.6649	y = −0.0077x − 802.72 R^2^ = 0.1081
Ankle z	y = − 7E-19x^6^ + 2E-14x^5^ − 2E-10x^4^ + 8E-07x^3^ − 0.0016x^2^ + 1.1437x + 2440.4 R^2^ = 0.8088	y = 0.0499x + 2428.1 R^2^ = 0.3469	ankle z	y = 8E-19x^6^ − 2E-14x^5^ + 2E-10x^4^ − 6E-07x^3^ + 0.001x^2^ − 0.6664x + 2706.6 R^2^ = 0.7414	y = 0.0307x + 2507.5 R^2^ = 0.2011

**Table 4:** Acceleration showing maximum and minimum points to various angle positions, non-dancers.

Column Name	Time (m/s) Min	Time (m/s) Max	Acceleration of Min, mm/s^2^	Acceleration of Max, mm/s^2^
ShoulderLeft x (mm)	3.674	2.407	−0.118	0.294
ShoulderLeft y (mm)	4.517	4.039	0.237	0.43
ShoulderLeft z (mm)	3.875	5.639	2.614	2.947
ElbowLeft x (mm)	3.422	2.374	−0.264	0.093
ElbowLeft y (mm)	6.238	2.738	0.054	0.45
ElbowLeft z (mm)	4.006	5.506	2.395	2.997
ElbowRight x (mm)	0.014	5.605	0.359	0.758
ElbowRight y (mm)	0.014	5.338	0.05	0.432
ElbowRight z (mm)	2.773	5.273	2.34	2.863
ShoulderRight x (mm)	4.174	2.24	0.215	0.612
ShoulderRight y (mm)	2.139	5.273	0.229	0.427
ShoulderRight z (mm)	2.607	7.275	2.526	2.89
HipLeft x (mm)	3.674	2.308	−0.035	0.376
HipLeft y (mm)	4.517	5.238	−0.23	−0.056
HipLeft z (mm)	2.674	7.139	2.593	2.855
KneeLeft x (mm)	3.638	2.674	−0.052	0.33
KneeLeft y (mm)	6.81	2.81	−0.561	−0.318
KneeLeft z (mm)	2.607	7.405	2.329	2.806
AnkleLeft x (mm)	3.121	2.674	−0.103	0.452
AnkleLeft y (mm)	6.474	2.073	−0.897	−0.642
AnkleLeft z (mm)	2.207	7.308	2.194	2.786
HipRight x (mm)	3.674	2.308	0.132	0.528
HipRight y (mm)	4.517	5.206	−0.228	−0.045
HipRight z (mm)	2.674	7.34	2.561	2.839
KneeRight x (mm)	4.039	2.106	0.175	0.532
KneeRight y (mm)	6.774	3.488	−0.562	−0.37
KneeRight z (mm)	2.773	7.405	2.351	2.812
AnkleRight x (mm)	4.006	5.106	0.168	0.544
AnkleRight y (mm)	5.939	3.911	−0.892	−0.714
ElbowRight x (mm)	0.014	5.605	0.359	0.758

**Table 5:** Acceleration showing maximum and minimum points to various angle positions dancers.

Column Name	Time (m/s^2^) of Min	Time (m/s^2^) of Max	Acceleration of Min mm/s^2^	Acceleration of Max, mm/s^2^
ShoulderLeft x (mm)	0.908	4.308	−0.426	−0.139
ShoulderLeft y (mm)	1.942	3.542	0.736	0.787
ShoulderLeft z (mm)	4.308	0.012	1.732	3.952
ElbowLeft x (mm)	0.908	4.308	−0.5	−0.207
ElbowLeft y (mm)	2.007	3.704	0.47	0.561
ElbowLeft z (mm)	4.308	0.407	1.763	3.951
ShoulderRight x (mm)	0.874	4.308	−0.1	0.196
ShoulderRight y (mm)	1.942	3.643	0.733	0.781
ShoulderRight z (mm)	4.308	0.206	1.717	3.991
ElbowRight x (mm)	1.003	4.308	−0.048	0.263
ElbowRight y (mm)	1.341	4.271	0.47	0.541
ElbowRight z (mm)	4.308	0.407	1.696	3.983
HipLeft x (mm)	0.874	4.308	−0.35	−0.064
HipLeft y (mm)	2.574	0.012	0.265	0.304
HipLeft z (mm)	4.308	0.012	1.731	3.969
KneeLeft x (mm)	0.874	4.308	−0.365	−0.1
KneeLeft y (mm)	2.642	1.842	−0.128	−0.02
KneeLeft z (mm)	4.308	0.241	1.671	3.932
AnkleLeft x (mm)	0.774	4.308	−0.348	−0.122
AnkleLeft y (mm)	4.07	0.569	−0.458	−0.395
AnkleLeft z (mm)	4.308	0.44	1.751	3.964
HipRight x (mm)	0.874	4.308	−0.204	0.083
HipRight y (mm)	1.87	2.941	0.258	0.302
HipRight z (mm)	4.308	0.012	1.72	3.983
KneeRight x (mm)	0.774	4.308	−0.167	0.087
KneeRight y (mm)	3.275	2.107	−0.135	−0.038
KneeRight z (mm)	4.308	0.407	1.727	3.971
AnkleRight x (mm)	0.84	4.241	−0.147	0.062
AnkleRight y (mm)	2.342	2.107	−0.466	−0.331
AnkleRight z (mm)	4.07	0.34	1.724	4.009

**Table 6:** The table below showing regression equation and slopes.

Left	Poly-Regression Equation	Slope	Right	Poly-Regression Equation	Slope
Shoulder x	y = −5E-14x^6^ + 2E-11x^5^ − 4E-09x^4^ + 3E-07x^3^ − 1E-05x^2^ + 0.0002x − 0.0005 R^2^ = 0.0017	y = −3E-06x + 0.0003 R^2^ = 0.0005	shoulder x	y = 8E-12x^6^ − 3E-09x^5^ + 5E-07x^4^ − 4E-05x^3^ + 0.0013x^2^ − 0.019x + 0.0588 R^2^ = 0.0271	y = 0.0001x + 0.0008 R^2^ = 0.0015
Shoulder y	y = 9E-14x^6^ − 4E-11x^5^ + 5E-09x^4^ − 4E-07x^3^ + 1E-05x^2^ − 0.0002x + 0.0007 R^2^ = 0.0077	y = −3E-06x + 0.0003 R^2^ = 0.0005	shoulder y	y = 7E-12x^6^ − 2E-09x^5^ + 2E-07x^4^ − 2E-06x^3^ − 0.0006x^2^ + 0.0262x − 0.107 R^2^ = 0.1474	y = −0.0009x + 0.1613 R^2^ = 0.0353
Elbow x	y = 2E-12x^6^ − 9E-10x^5^ + 2E-07x^4^ − 2E-05x^3^ + 0.0007x^2^ − 0.0127x + 0.0391 R^2^ = 0.0226	y = 0.0002x − 0.0055 R^2^ = 0.0048	elbow x	y = 5E-14x^6^ − 2E-11x^5^ + 2E-09x^4^ − 2E-07x^3^ + 5E-06x^2^ − 7E-05x + 0.0006 R^2^ = 0.0019	y = −1E-06x + 0.0002 R^2^ = 5E-05
Elbow y	y = −4E-14x^6^ + 2E-11x^5^ − 3E-09x^4^ + 3E-07x^3^ − 1E-05x^2^ + 0.0001x − 7E-05 R^2^ = 0.0018	y = −3E-06x + 0.0002 R^2^ = 0.0003	elbow y	y = 1E-11x^6^ − 4E-09x^5^ + 6E-07x^4^ − 4E-05x^3^ + 0.0013x^2^ − 0.016x + 0.039 R^2^ = 0.0304	y = −4E-05x + 0.0086 R^2^ = 1E-04
Hip x	y = 5E-14x^6^ − 2E-11x^5^ + 3E-09x^4^ − 2E-07x^3^ + 6E-06x^2^ − 9E-05x + 0.0005 R^2^ = 0.0033	y = −3E-07x + 8E-05 R^2^ = 3E-05	hip x	y = −4E-14x^6^ + 2E-11x^5^ − 3E-09x^4^ + 2E-07x^3^ − 9E-06x^2^ + 0.0001x − 0.0001 R^2^ = 0.0037	y = −3E-06x + 0.0002 R^2^ = 0.0012
Hip y	y = 5E-14x^6^ − 2E-11x^5^ + 3E-09x^4^ − 2E-07x^3^ + 6E-06x^2^ − 9E-05x + 0.0005 R^2^ = 0.0033	y = −3E-07x + 8E-05 R^2^ = 3E-05	hip y	y = 4E-14x^6^ − 2E-11x^5^ + 2E-09x^4^ − 1E-07x^3^ + 5E-06x^2^ − 6E-05x + 0.0003 R^2^ = 0.0035	y = 2E-07x + 4E-05 R^2^ = 2E-05
Knee x	y = −4E-15x^6^ + 1E-12x^5^ − 5E-12x^4^ − 2E-08x^3^ + 2E-06x^2^ − 5E-05x + 0.0003 R^2^ = 0.0001	y = 4E-07x − 4E-05 R^2^ = 7E-06	knee x	y = 4E-14x^6^ − 2E-11x^5^ + 3E-09x^4^ − 2E-07x^3^ + 8E-06x^2^ − 0.0001x + 0.0008 R^2^ = 0.0005	y = −3E-06x + 0.0002 R^2^ = 0.0002
Knee y	y = −4E-15x^6^ + 1E-12x^5^ − 5E-12x^4^ − 2E-08x^3^ + 2E-06x^2^ − 5E-05x + 0.0003 R^2^ = 0.0001	y = 4E-07x − 4E-05 R^2^ = 7E-06	knee y	y = 4E-14x^6^ − 2E-11x^5^ + 3E-09x^4^ − 2E-07x^3^ + 1E-05x^2^ − 0.0002x + 0.0011 R^2^ = 0.0007	y = 9E-07x − 0.0001 R^2^ = 2E-05
Ankle x	y = 3E-14x^6^ − 1E-11x^5^ + 1E-09x^4^ − 5E-08x^3^ + 4E-07x^2^ + 1E-07x + 0.0007 R^2^ = 0.0022	y = −6E-06x + 0.0005 R^2^ = 0.001	ankle x	y = 2E-14x^6^ − 7E-12x^5^ + 5E-10x^4^ + 7E-09x^3^ − 2E-06x^2^ + 8E-05x − 0.0004 R^2^ = 0.0005	y = −1E-06x + 7E-05 R^2^ = 4E-05
Ankle y	y = −3E-14x^6^ + 1E-11x^5^ − 3E-09x^4^ + 3E-07x^3^ − 1E-05x^2^ + 0.0002x − 0.0009 R^2^ = 0.0002	y = −1E-06x + 0.0001 R^2^ = 7E-06	ankle y	y = −1E-14x^6^ − 2E-12x^5^ + 1E-09x^4^ − 2E-07x^3^ + 9E-06x^2^ − 0.0002x + 0.0005 R^2^ = 0.0002	y = 1E-06x − 0.0003 R^2^ = 4E-06

## Data Availability

The data generated in this study are not publicly available due to the project’s educational nature and the absence of established data-sharing resources.
